# Supplementary material for: Several phased siRNA annotation methods can frequently misidentify 24 nucleotide siRNA‐dominated PHAS loci
Source: Plant Direct. 2018 Dec 17;2(12):e00101. doi: 10.1002/pld3.101 (PMC6508839; doi:10.1002/pld3.101)
Supplement: Supplementary file 1 [file PLD3-2-e00101-s001.pdf]

Figure S1 - page 1

Eudicots

Citrus sinensis (Sweet Orange)

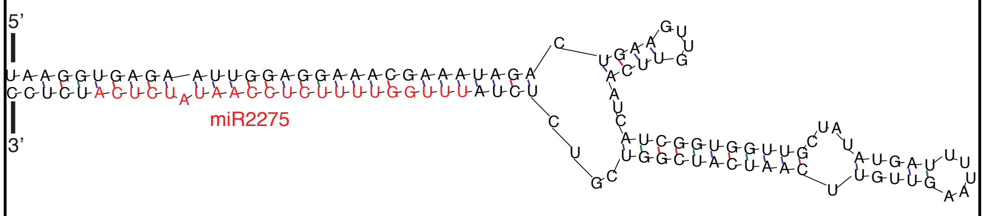

Scaffold02893:5,816-6,237

Citrus clementina (Clementine)

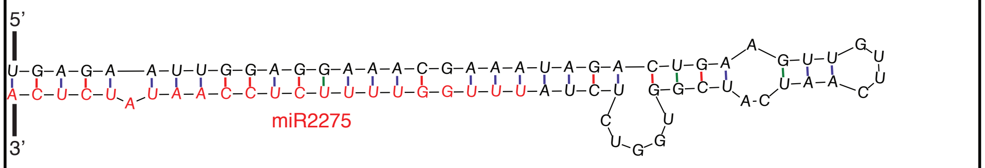

Scaffold_7:11,677,716-11,678,137

Populus trichocarpa (Black Cottonwood)

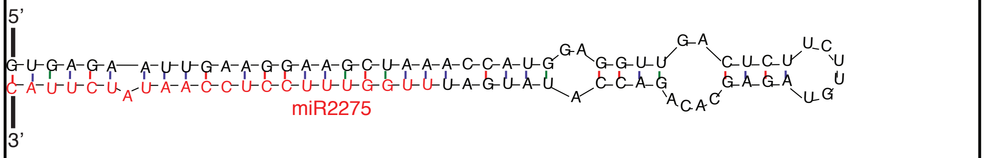

Chr18:16,602,794-16,603,213

Fragaria vesca (Woodland Stawberry)

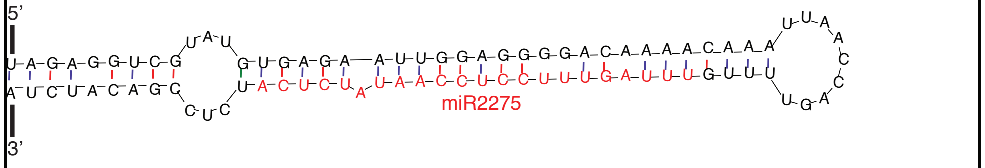

LG6:33,999,807-34,000,228

Figure S1 - page 2

Prunus persica (Peach)

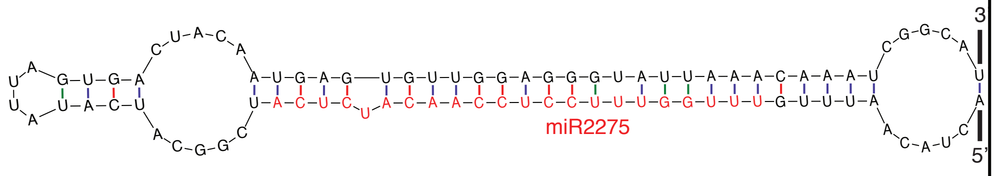

Pp03:25,091,557-25,091,979

Daucus carota (Carrot)

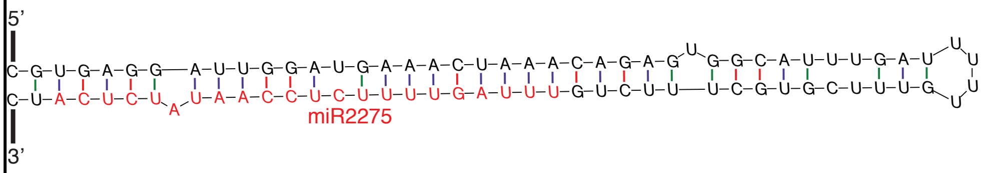

DCARv2_Chr3:50,126,000-50,126,421

Linum usitatissimum (Flax)

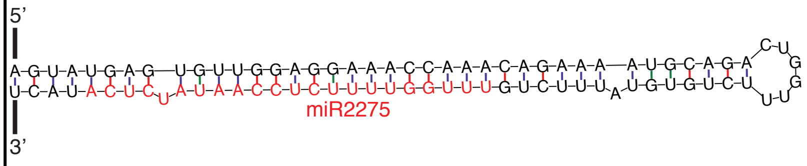

Scaffold917:325,461-325,882

Salix purpurea

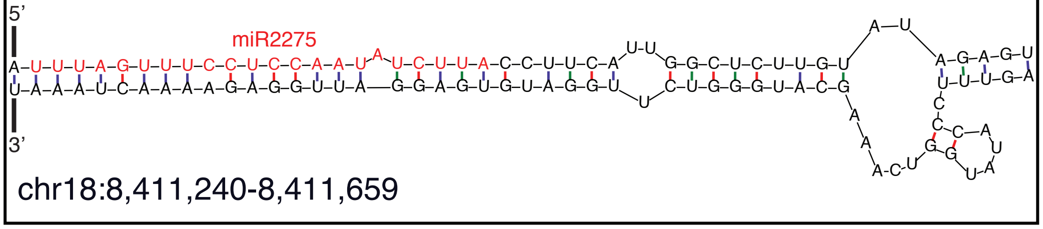

chr18:8,411,240-8,411,659

Figure S1 - page 3

Manihot esculenta(Cassava)

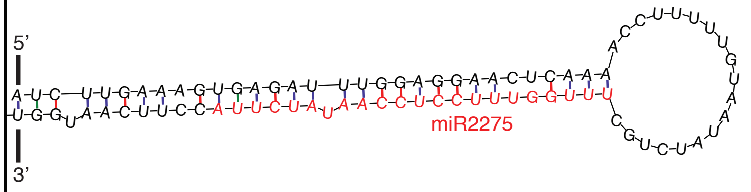

Chromosome09:25,243,068-25,243,487

Vitis vinifera(Grapevine)

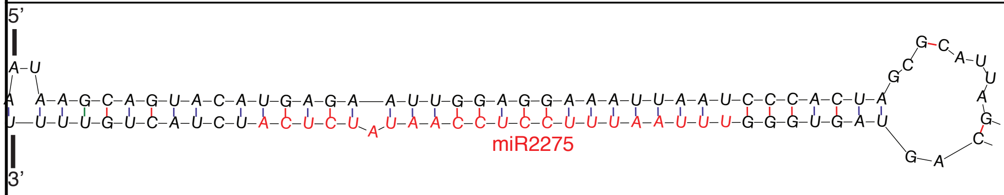

Chr1:11,599,187-11,599,609

Aquilegia coerulea

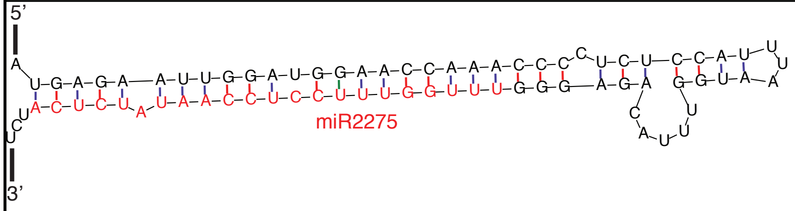

Chr5:8,028,124-8,028,545

Malus domestica(Apple)

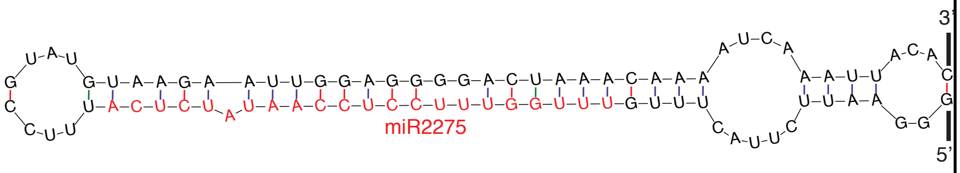

MDC013357.204:1,230-1,652

Figure S1 - page 4

Monocots

Panicum hallii

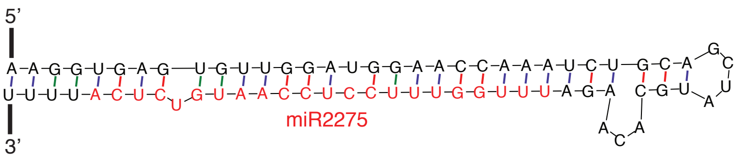

chr18_random:2,094,218-2,094,599

Ananas comosus (Pineapple)

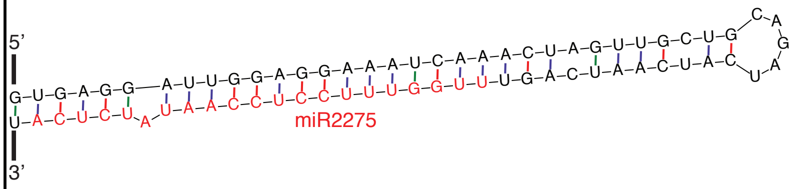

LG24:194,094-194,515

Brachypodium distachyon

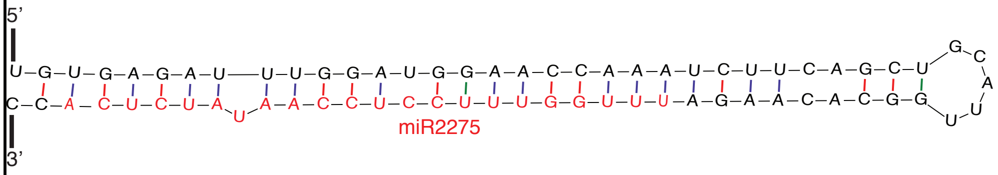

Bd3:12,534,229-12,534,651

Setaria viridis

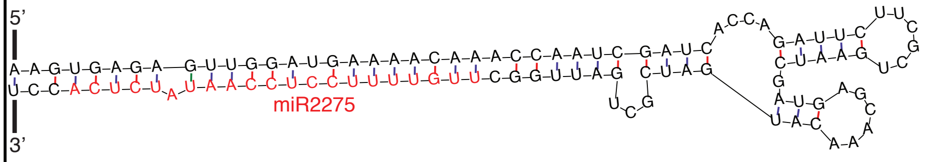

Chr_06:3,634,546-3,634,966

Figure S1 - page 5

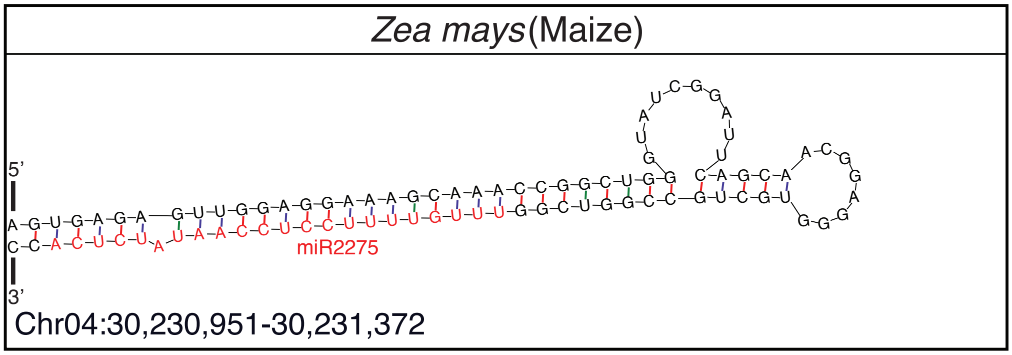

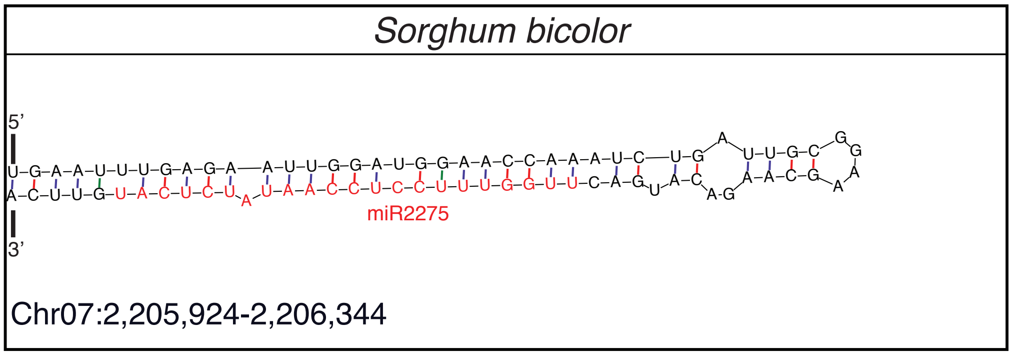

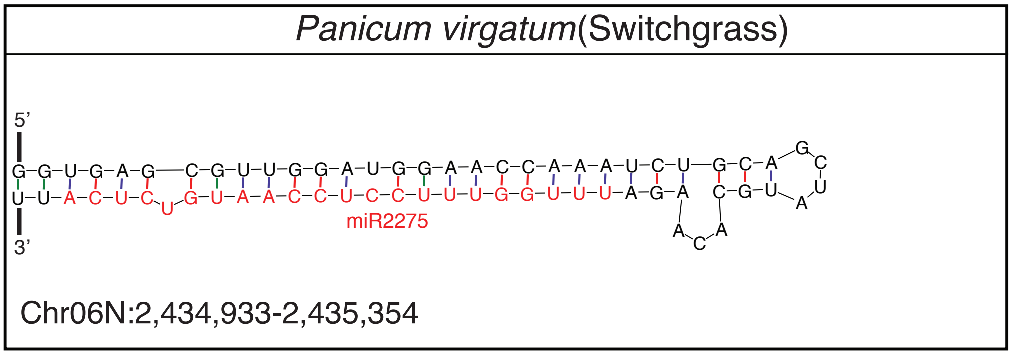

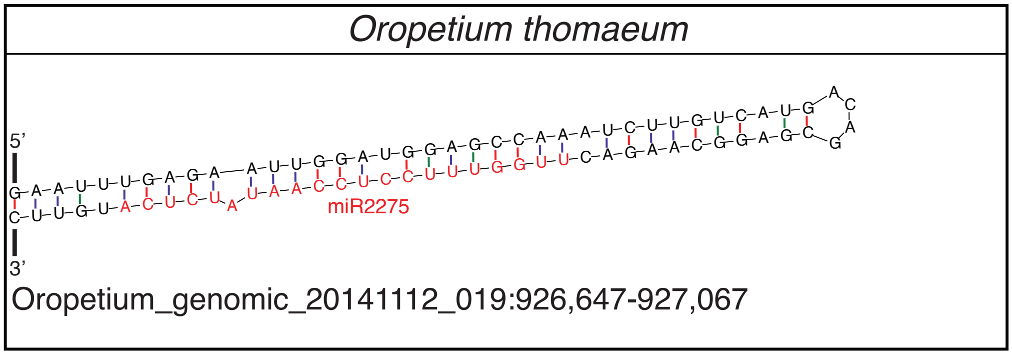

Figure S1 - page 6

Setaria italica

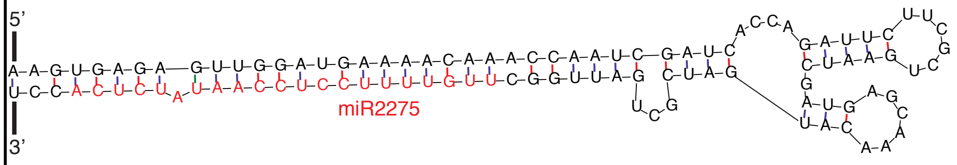

Scaffold_6:3,520,704-3,521,124

Figure S1. The RNA secondary structure of Bowtie-validated MIR2275 homologs. Secondary structures of loci were predicted via mFOLD. If the potential MIR2275 homolog formed a stem-and-loop structure, it is shown above. The red nucleotides denote the mature MIR2275 homolog in the secondary structure.

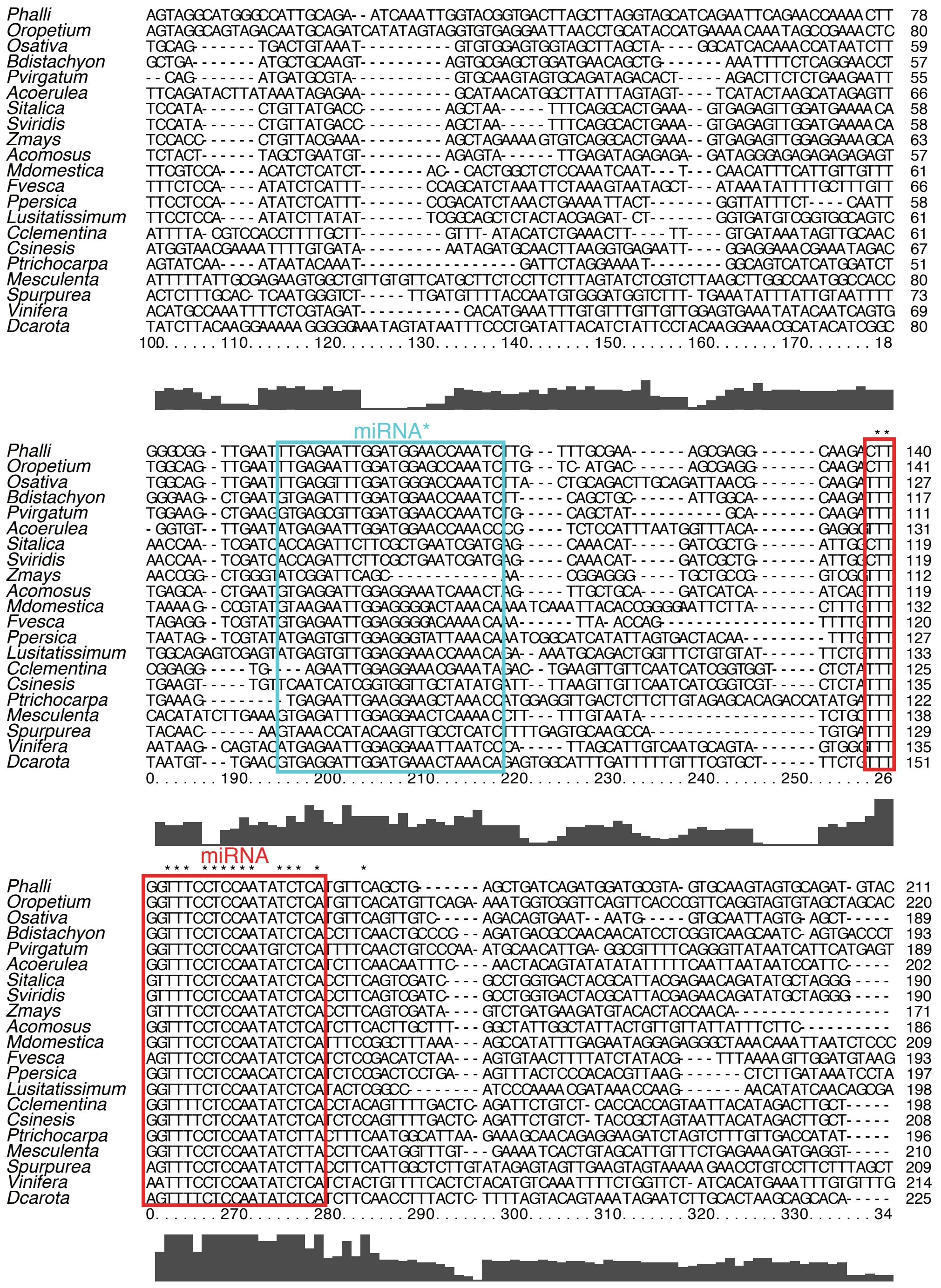

Figure S2. Alignment of MIR2275 homologs.
Sequences corresponding to miR2275 wih flanking 200 bp islands were extracted from genomes of the above-listed genomes and aligned via ClustalX2. Only the 100th to 340th nucleotide of each sequence (including gaps) are shown. miR2275 and miR2275* in each species are shown.

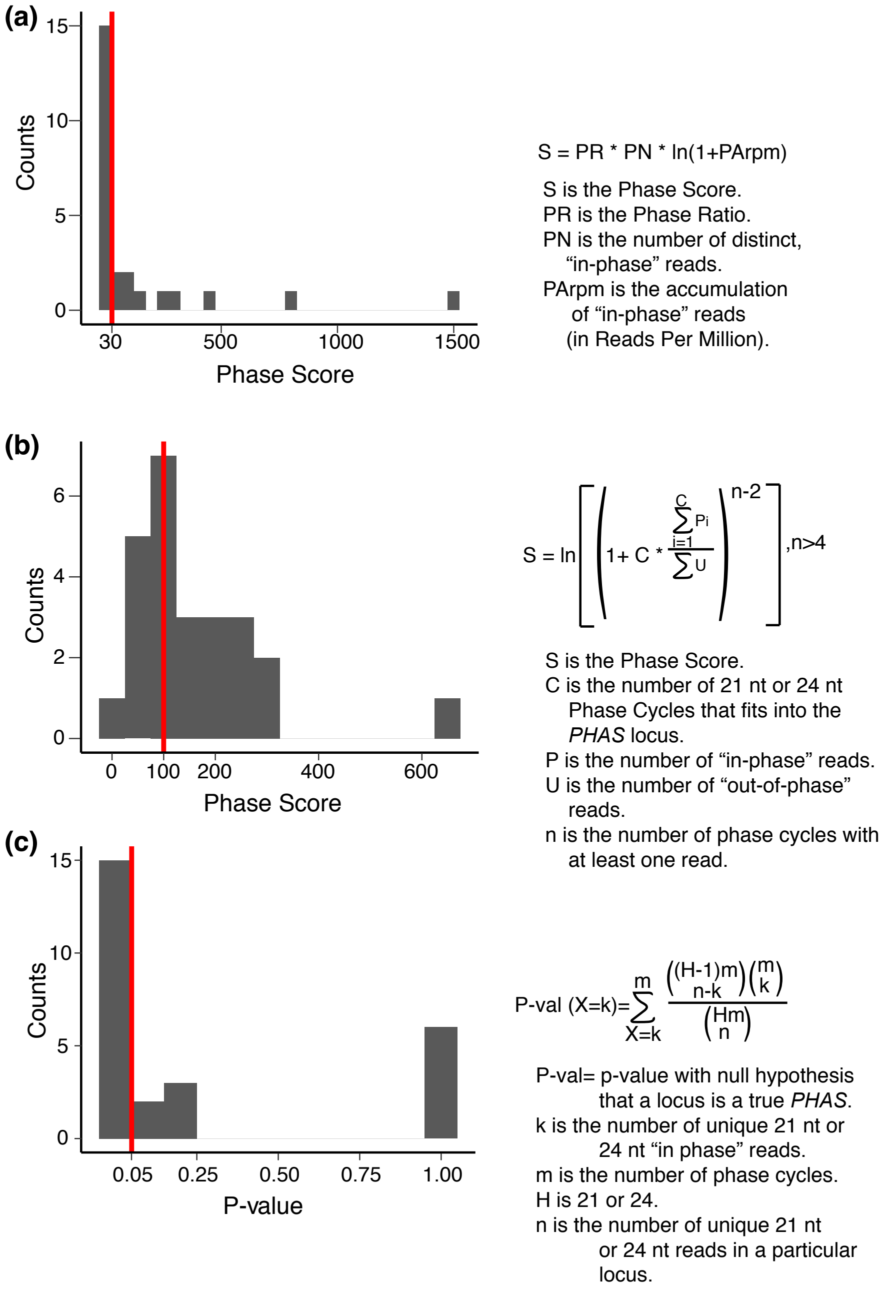

Figure S3. Determination of phase score cutoffs using three different algorithms
(a) Small RNA accumulation at 27 known 21 nt PHAS loci (Table S1) was analyzed using the PHAS-Test algorithm from Guo et al. (2015), which is used by ShortStack. Variables and formula are shown. Red line represents the cut-off used in this study to determine if a locus was phased or not; scores above the red line were considered 'phased'.
(b) Same as in panel a except for the PHAS-Test algorithm from Dotto et al. (2014).
(c) Same as in panel a except for the PHAS-Test algorithm from Zheng et al. (2014); scores below the red-line were considered 'phased'.

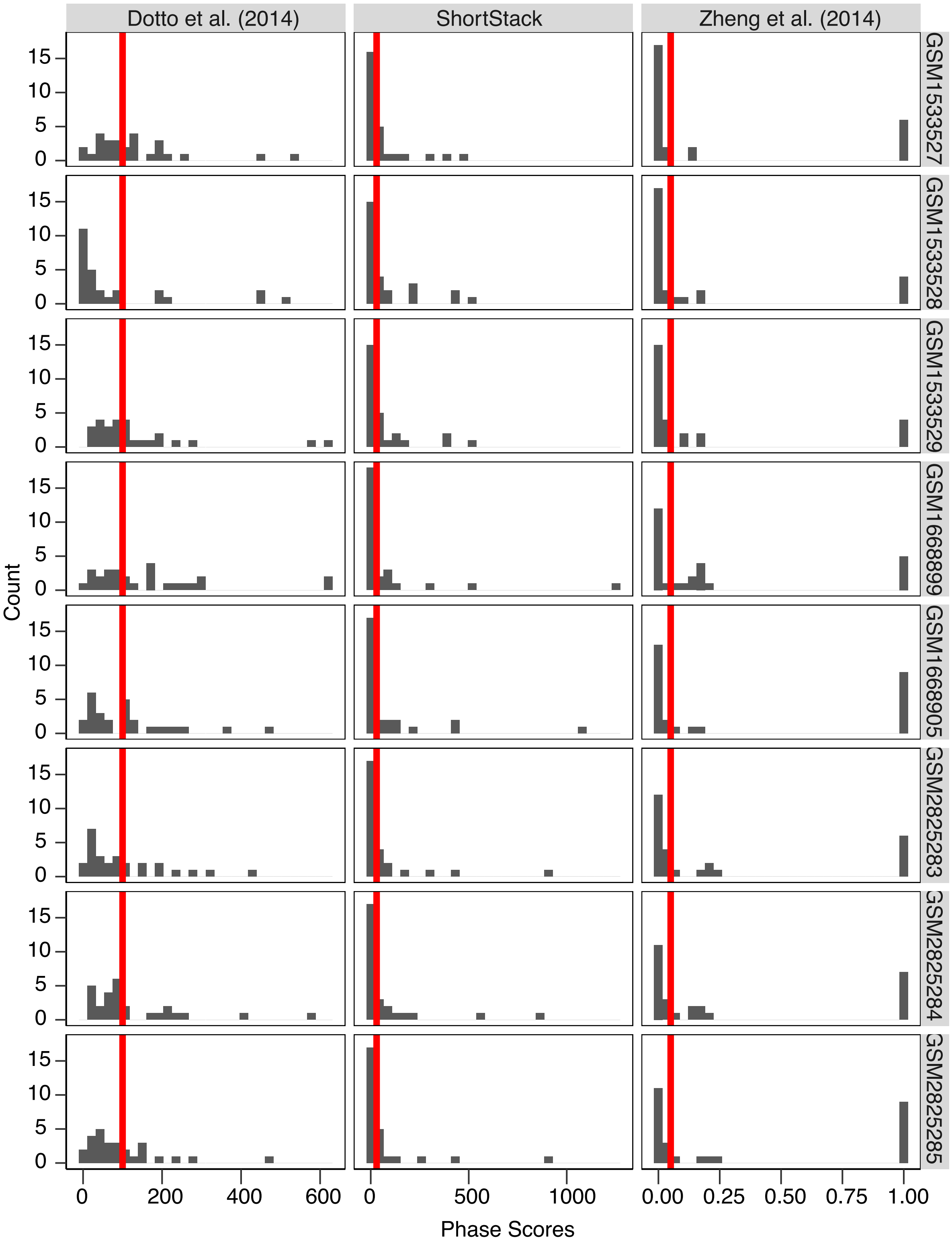

Figure S4. Phase scores of 27 previously known A. thaliana phased siRNA loci from the indicated algorithms. Cutoff values for called 'phasing' in our study are shown in red.

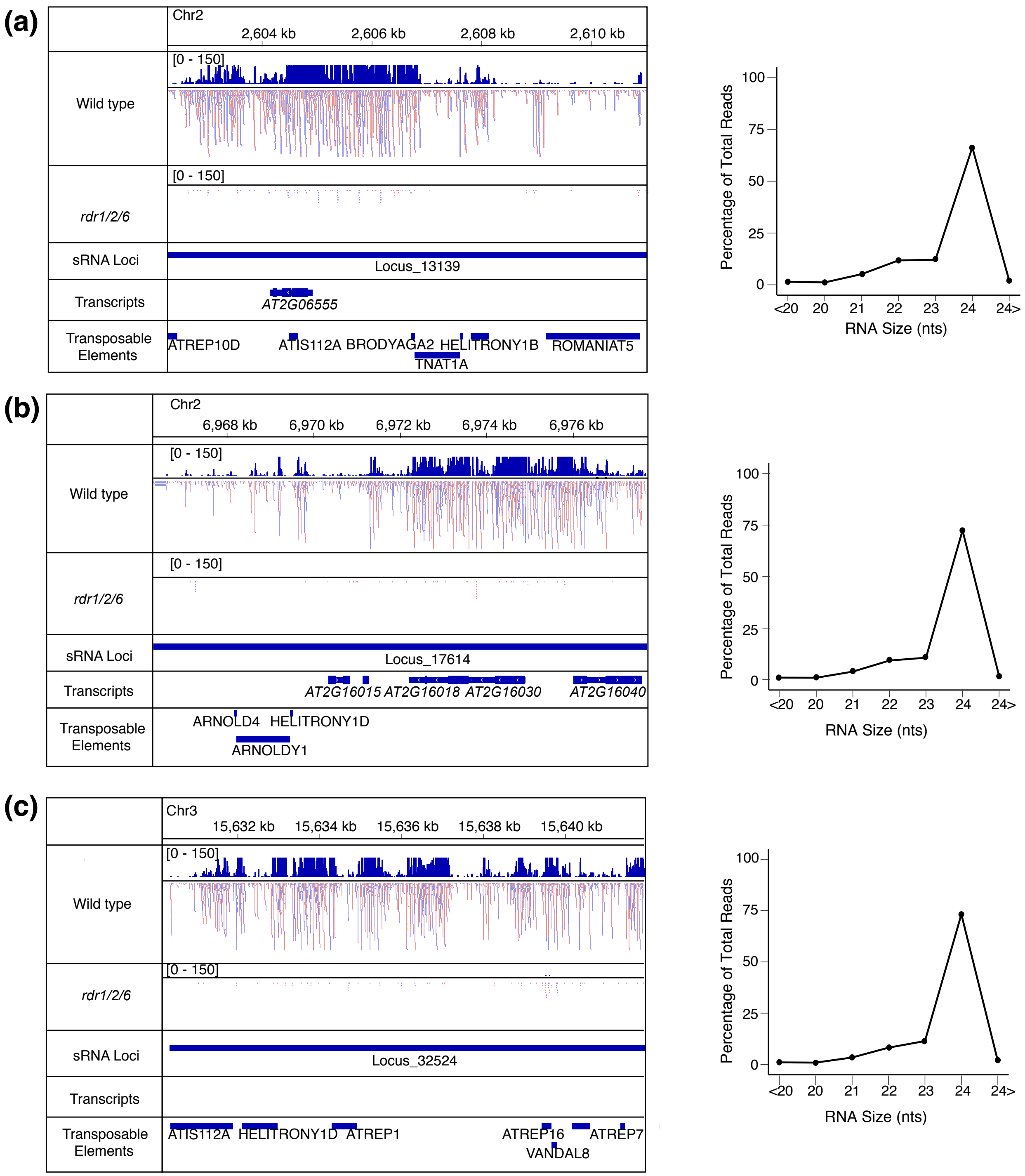

Figure S5. Genome browser snapshots of the three Arabidopsis thaliana PHAS-Test passing loci.
(a) Alignments and read size distribution of Locus_13139. Red reads represent positive-strand mapped genes, while blue reads are those that mapped to the negative strand. Numbers in the brackets are the range of coverage shown in Reads per Million.
(b) Same as a, except for Locus_17614.
(c) Same as a, except for Locus_32524.

Figure S6 - Page 1

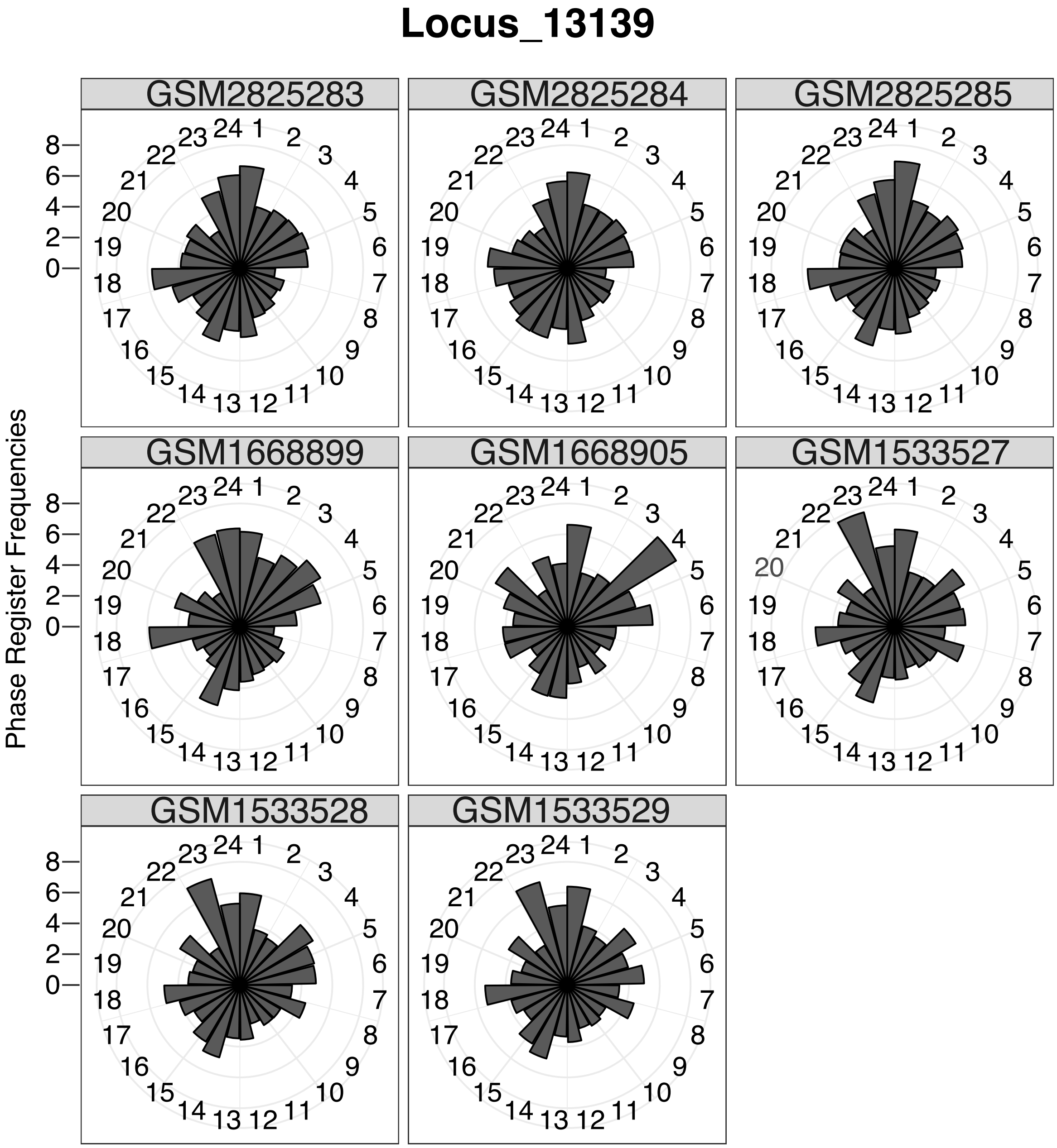

Figure S6 - Page 2

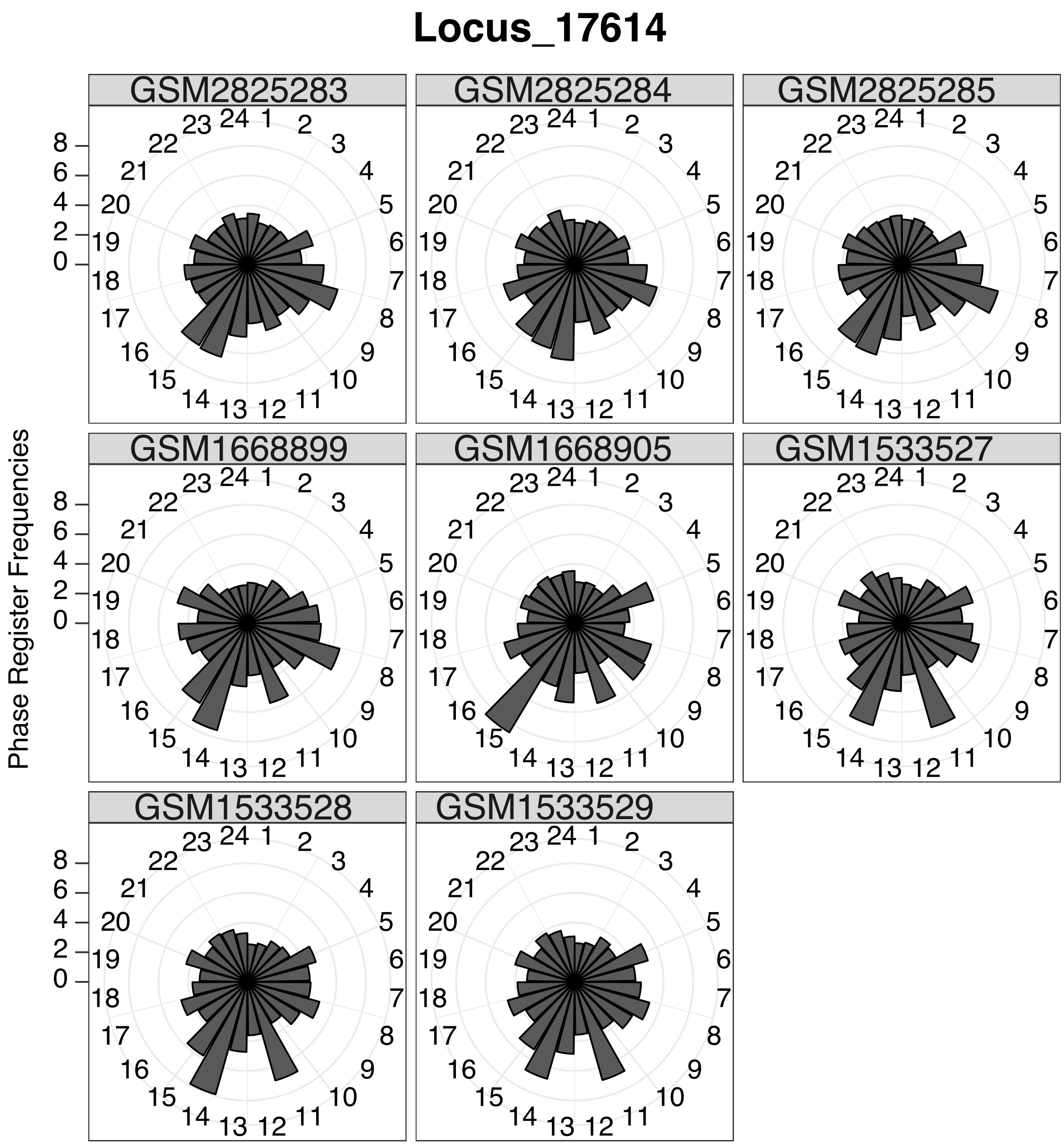

Figure S6 - Page 3

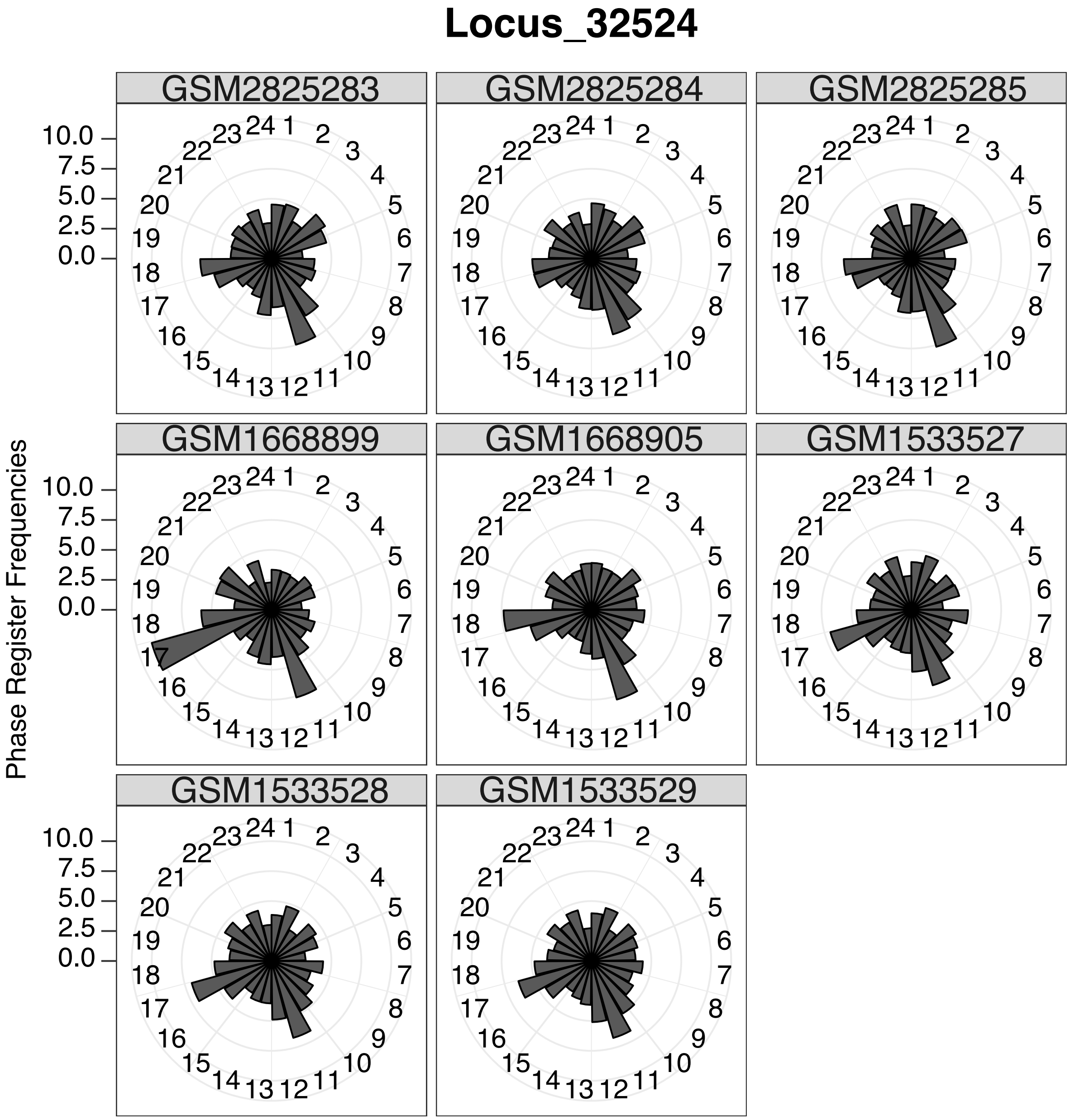

Figure S6 - Page 4

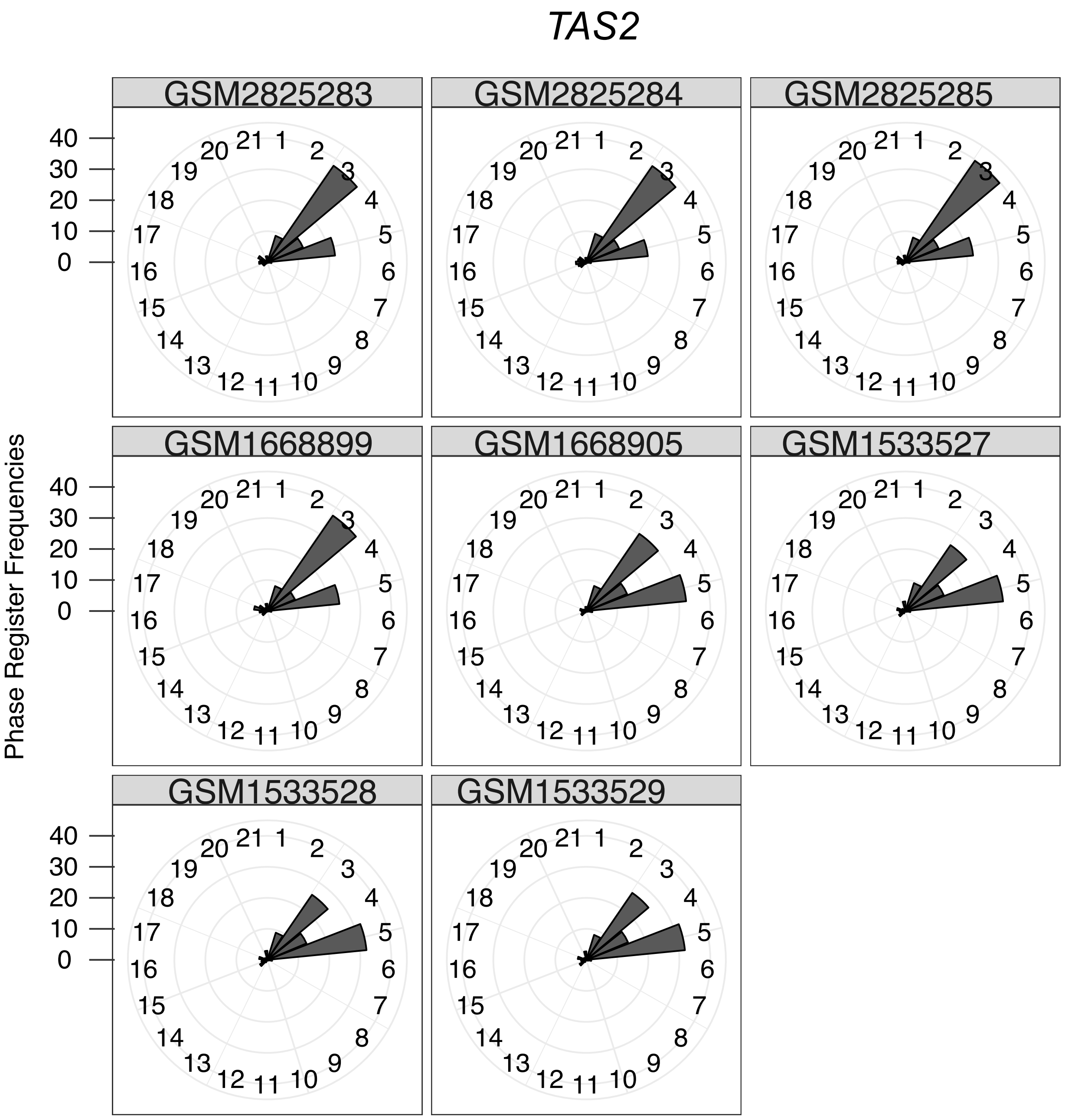

Figure S6. The three PHAS-Test passing loci have inconsistent phase register frequencies. Frequencies of phase registers where calculated in eight publicly available wild-type inflorescence libraries from A. thaliana (the accession numbers are listed in the grey boxes). TAS2 is shown for comparison. Phase register frequencies are calculated via the following formula: (number of reads “in phase”/ total number of reads at locus)*100.

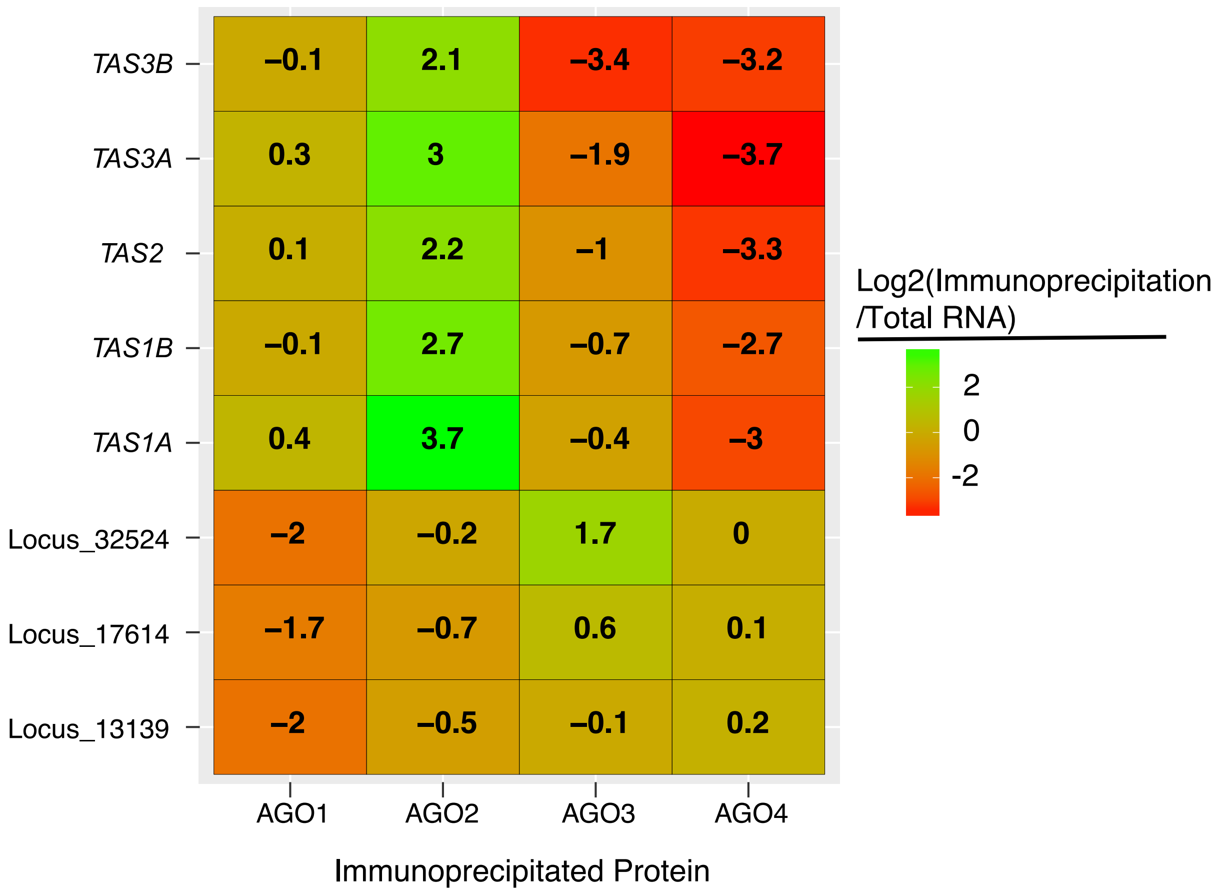

Figure S7. The three putative 24 nt PHAS loci show AGO-loading profiles that are distinct from TAS loci.
Small RNAs from immunoprecipitation protein were aligned to the A. thaliana (TAIR10) genome. Numbers indicate the ratio of sRNA accumulation between immunoprecipitated and total libraries (in RPMs).

Figure S8 - Page 1

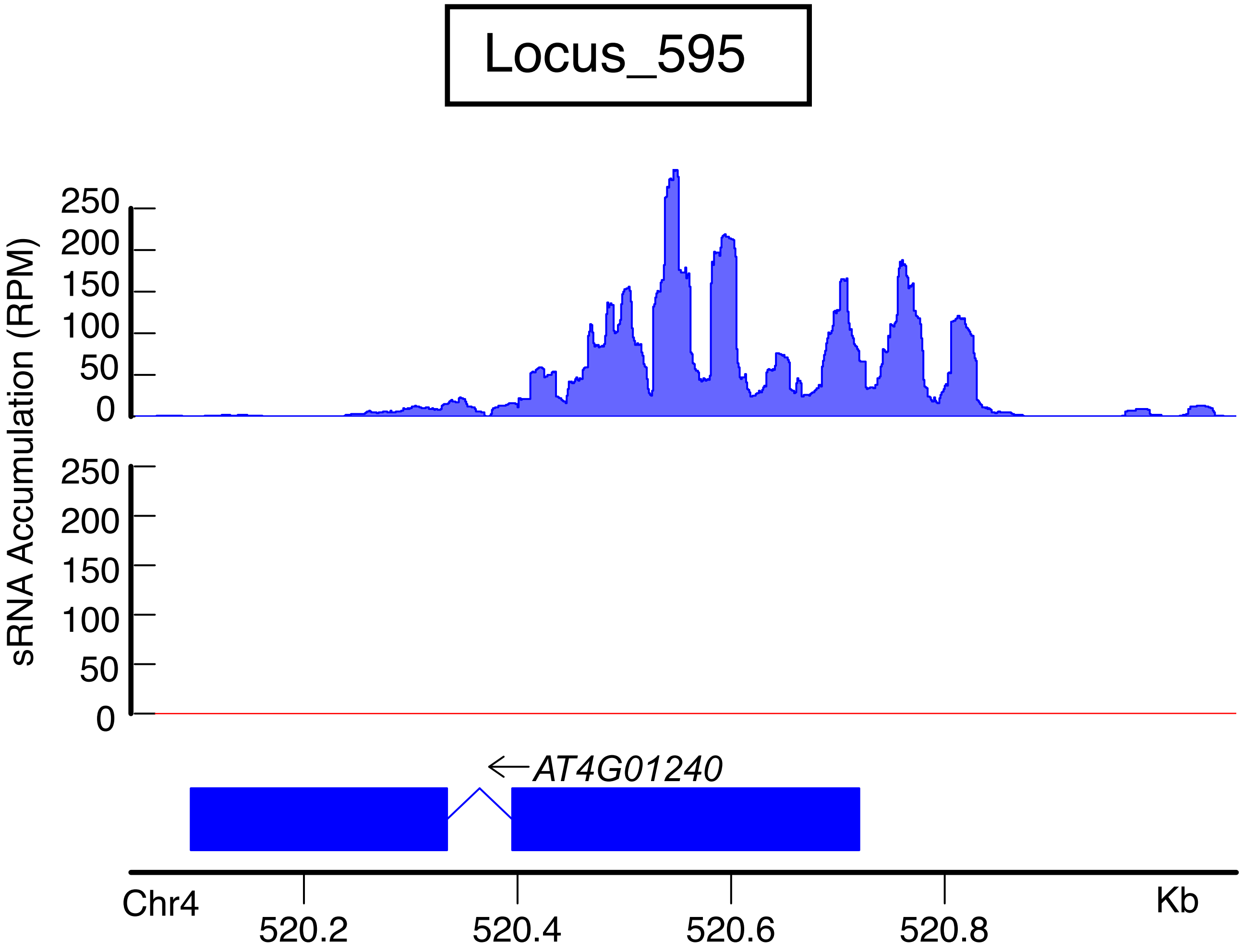

Figure S8 - Page 2

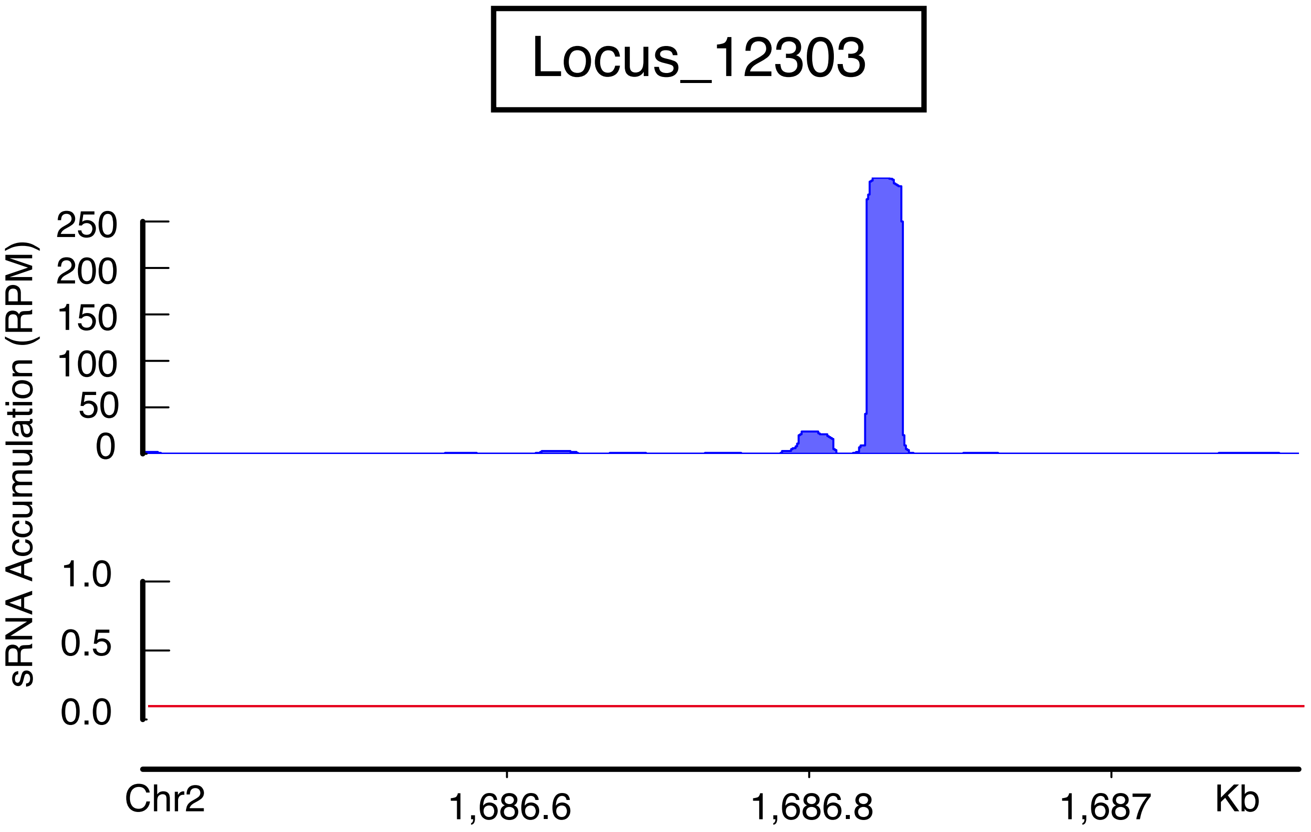

Figure S8 - Page 3

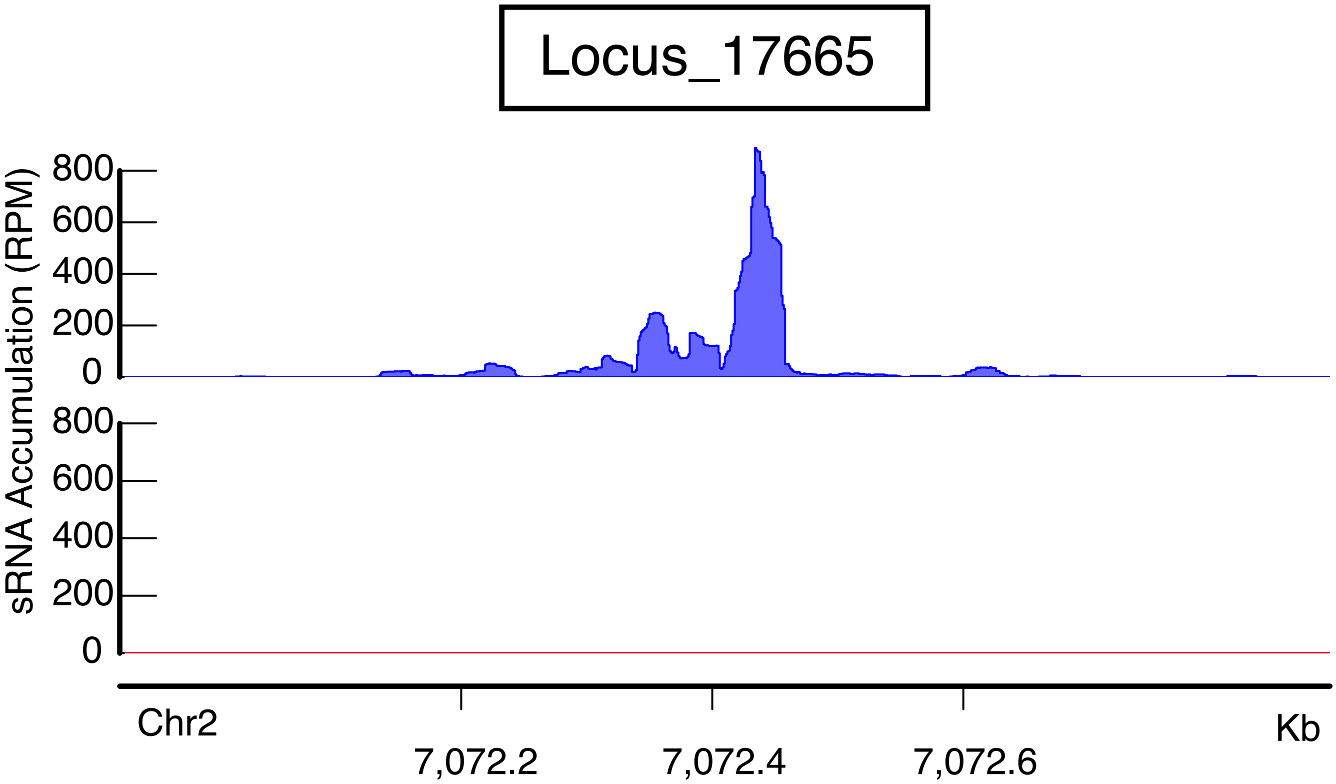

Figure S8 - Page 4

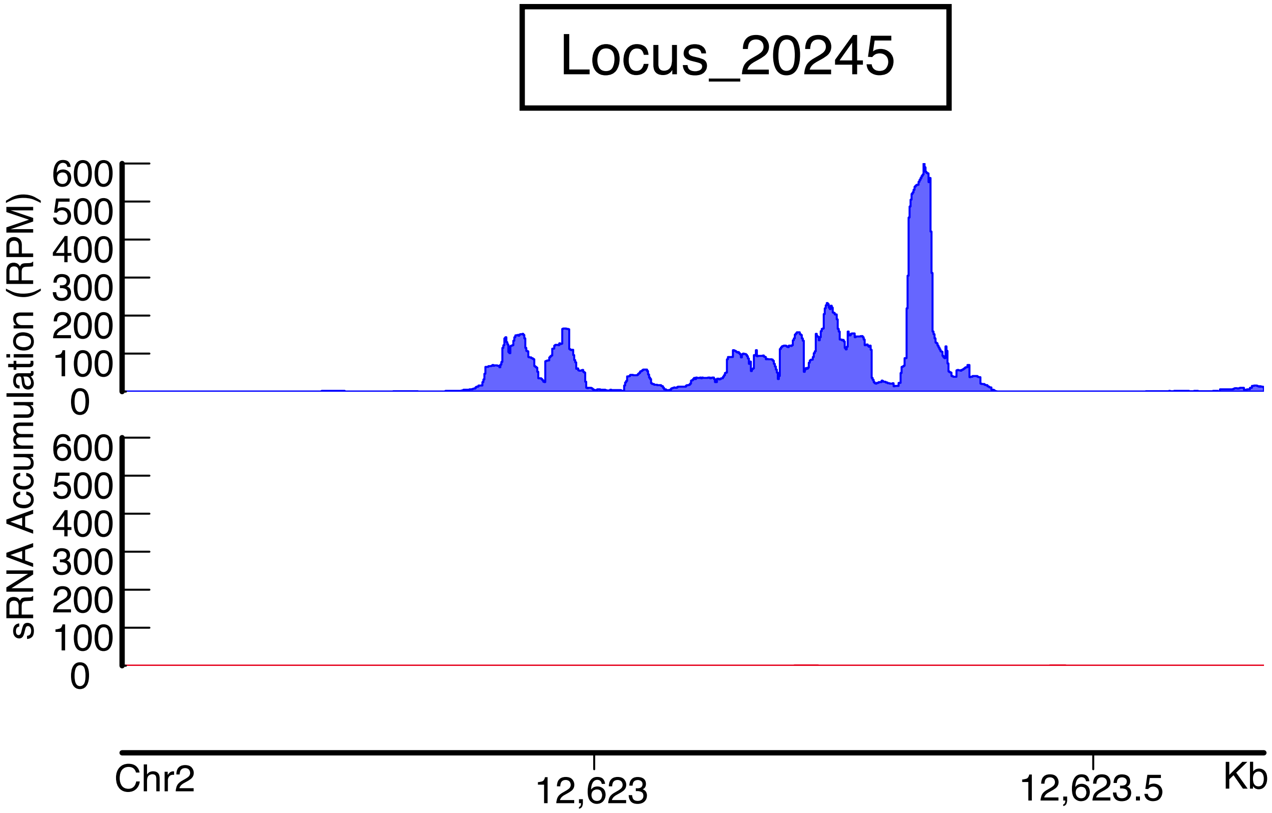

Figure S8 - Page 5

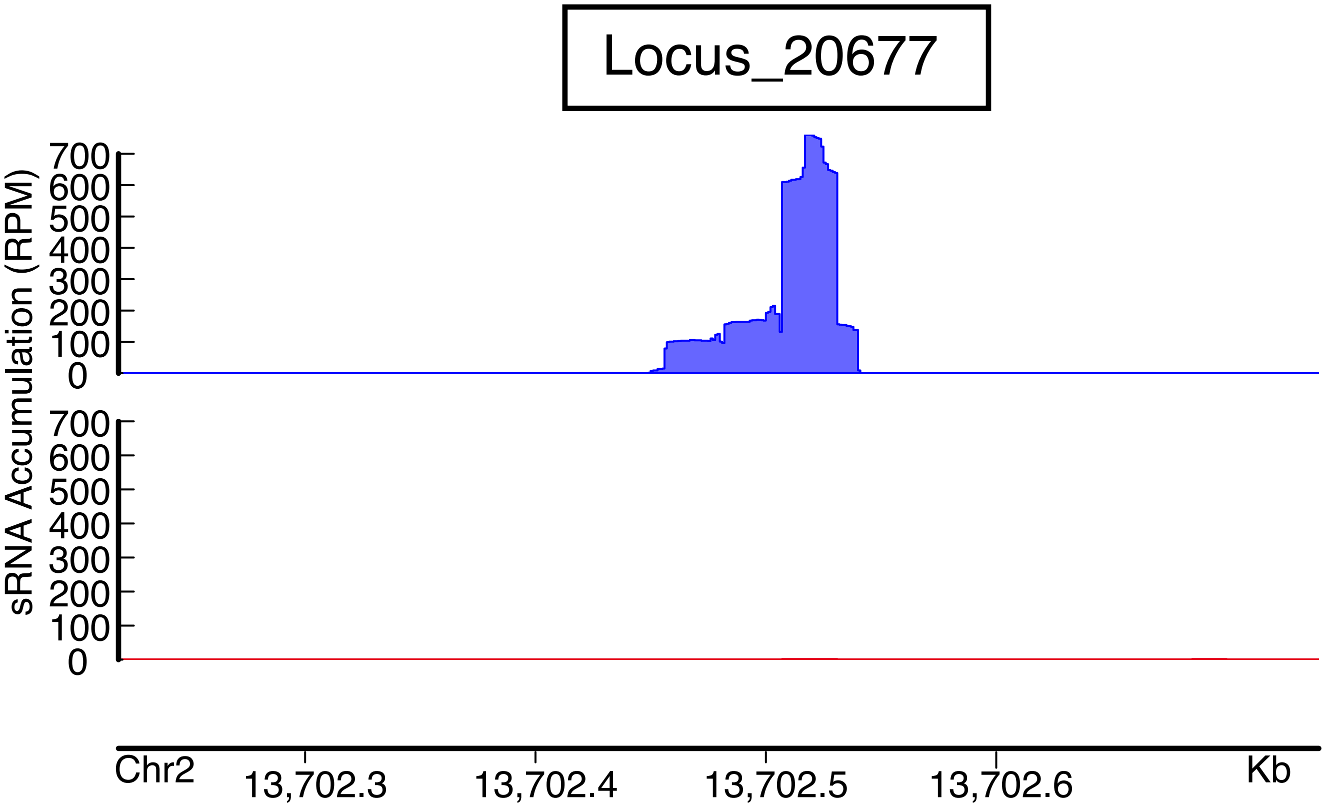

Figure S8 - Page 6

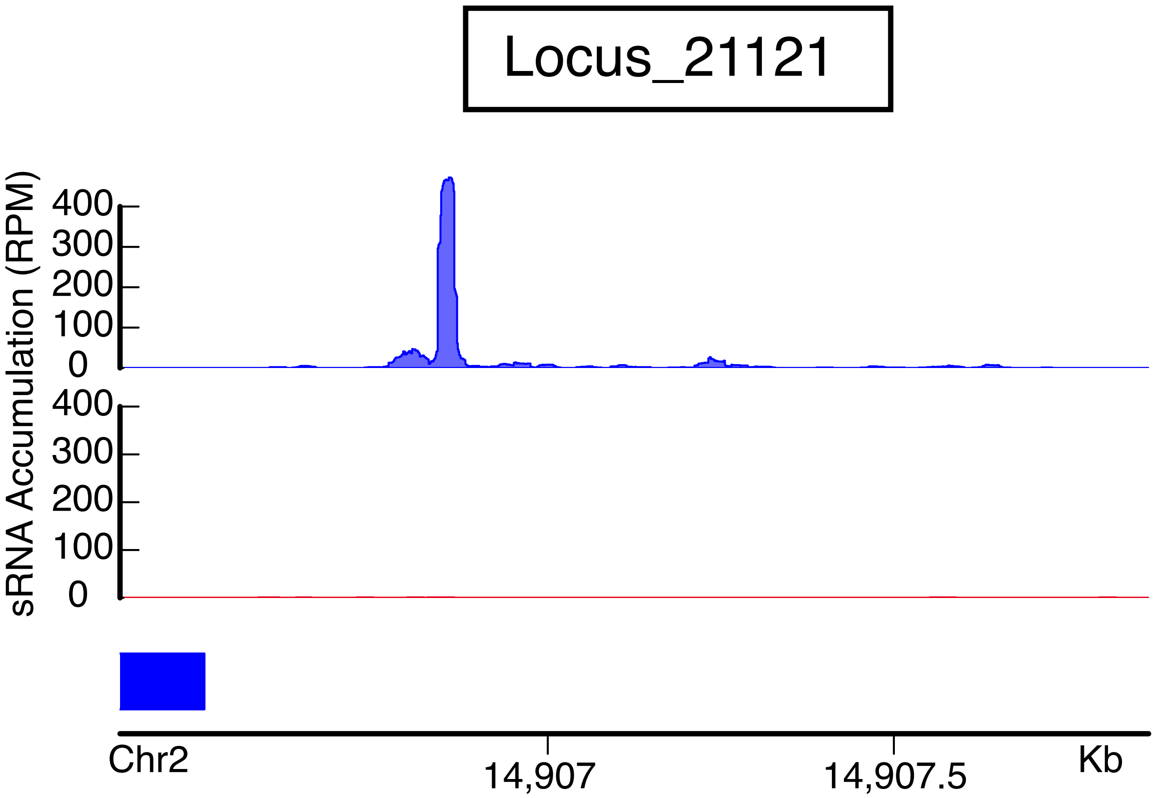

Figure S8 - Page 7

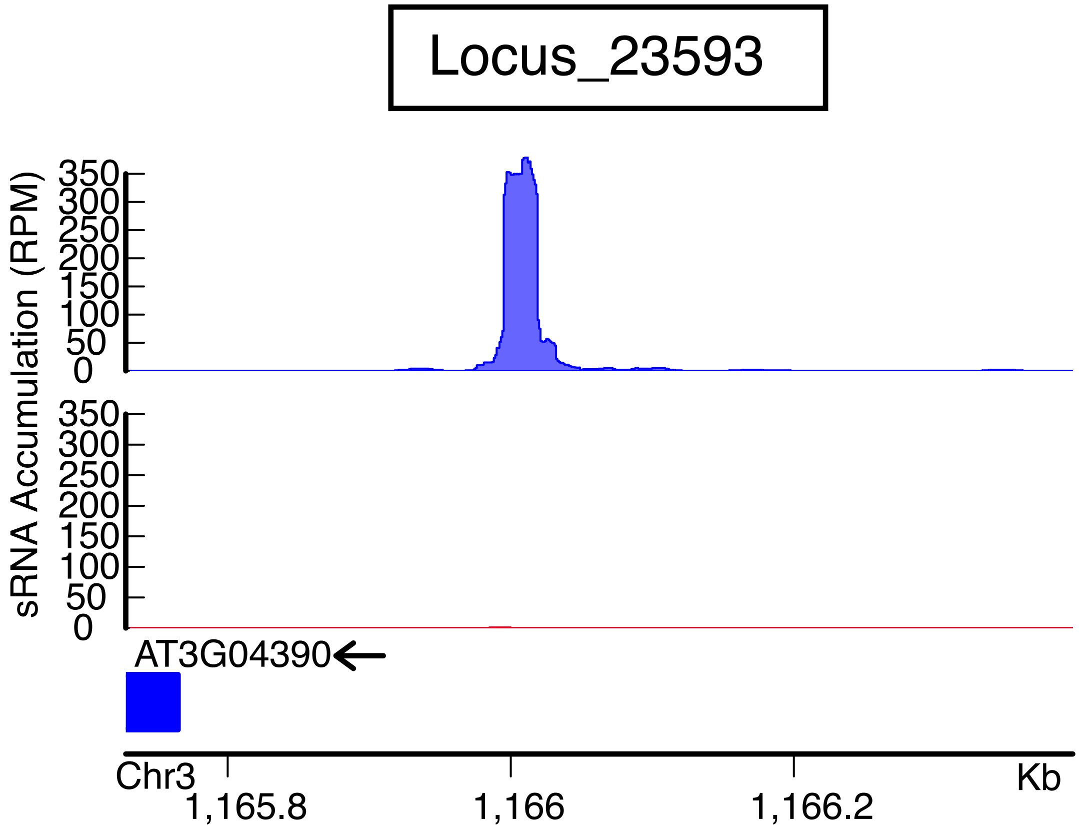

Figure S8 - Page 8

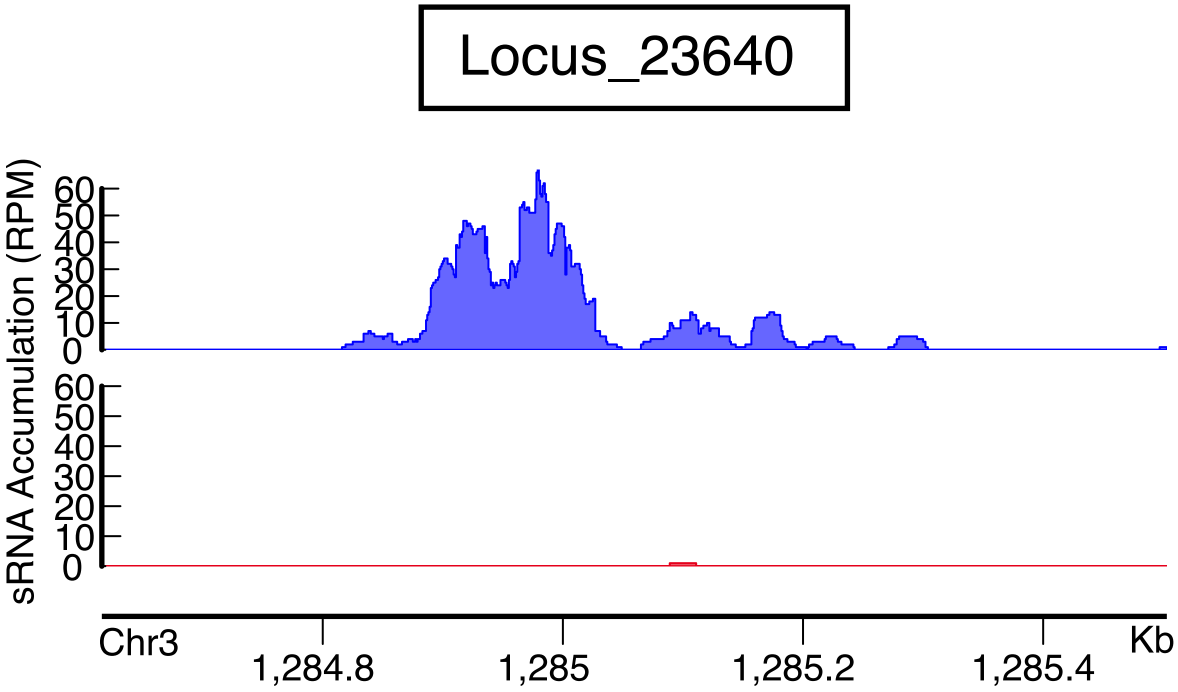

Figure S8 - Page 9

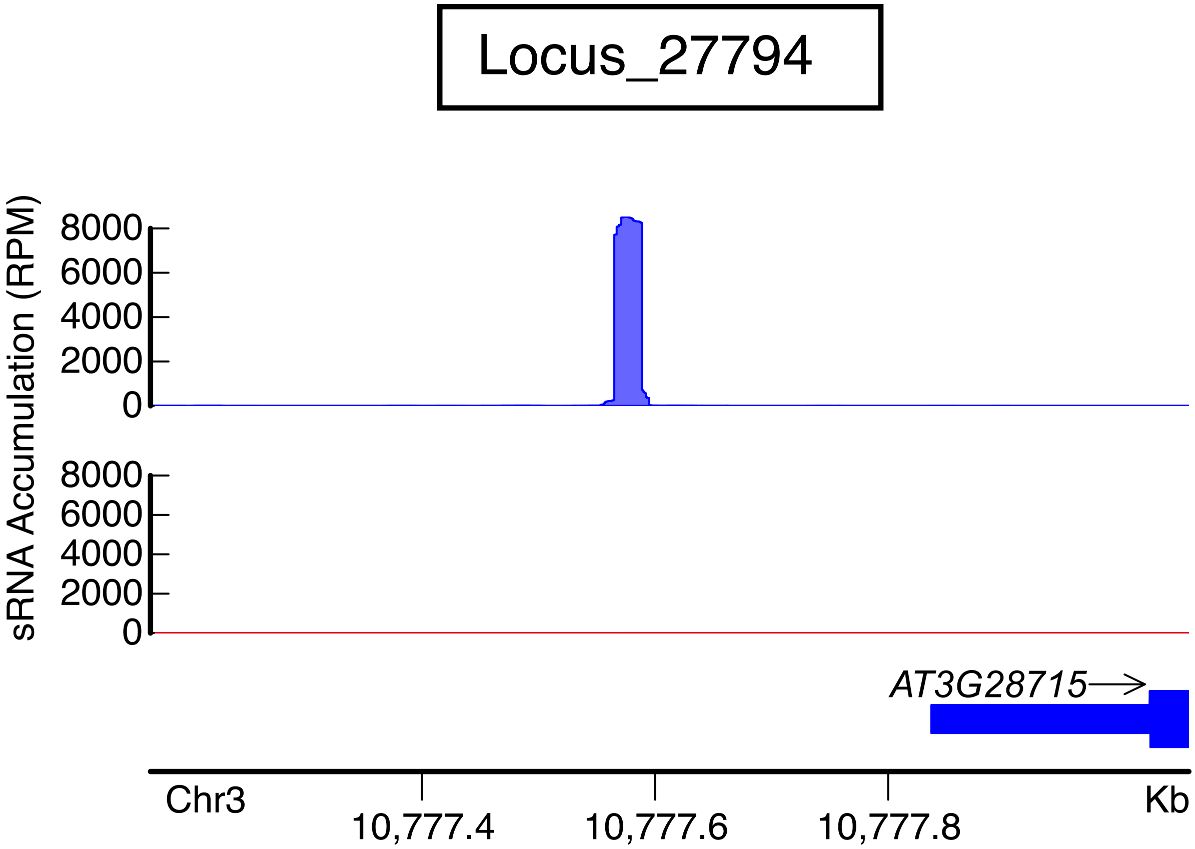

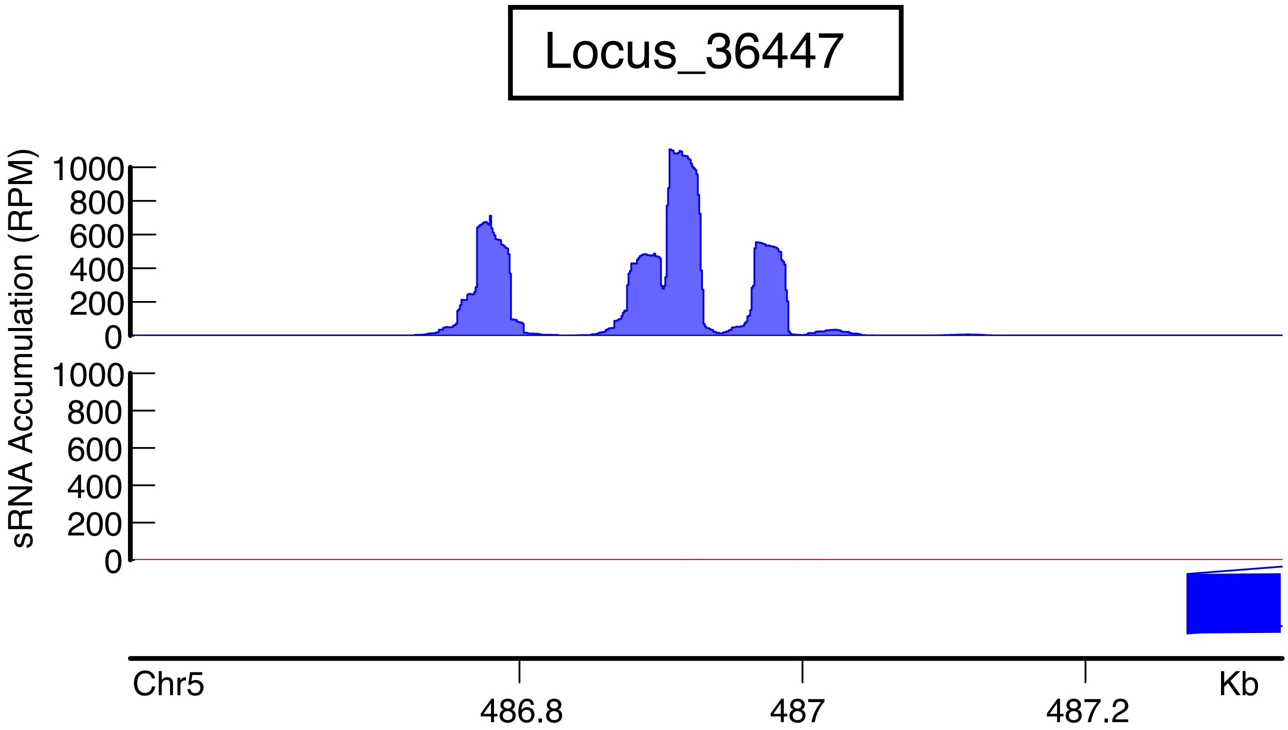
Locus_36447
sRNA Accumulation (RPM)
1000
800
600
400
200
0
1000
800
600
400
200
0
Chr5
486.8
487
487.2
Kb

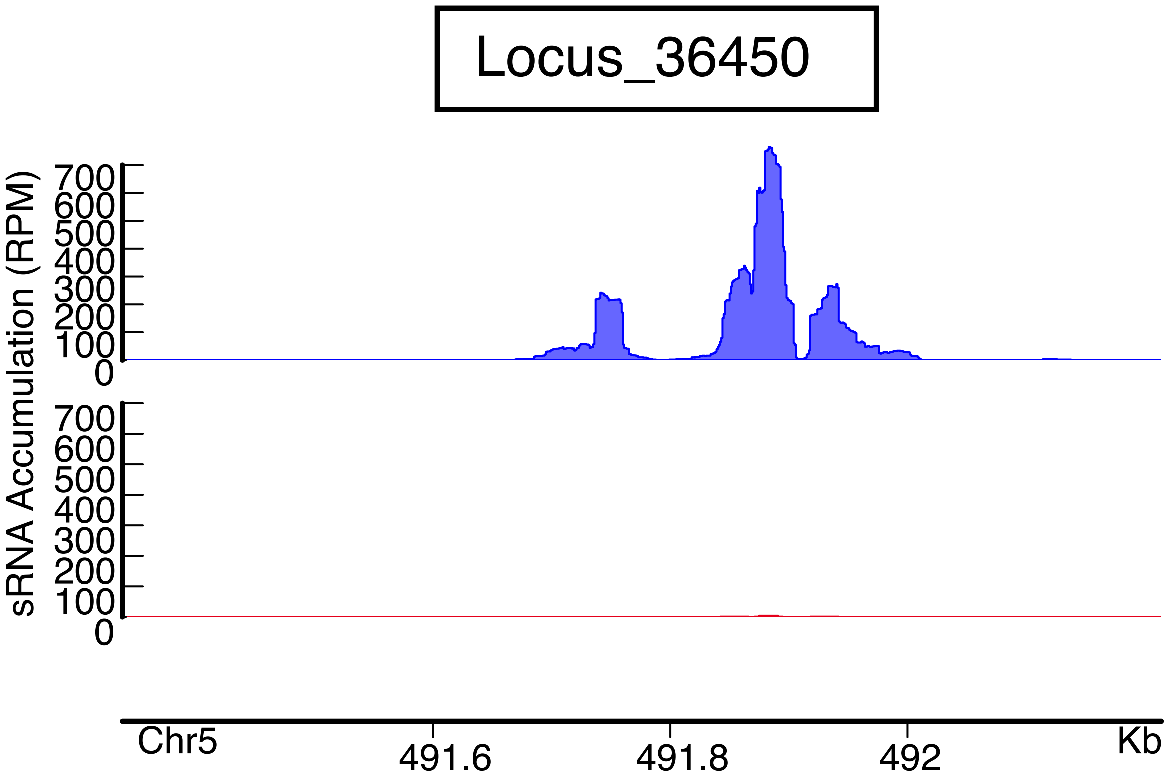
Locus_36450
sRNA Accumulation (RPM)
700
600
500
400
300
200
100
0
700
600
500
400
300
200
100
0
Chr5
491.6
491.8
492
Kb

Figure S8 - Page 12

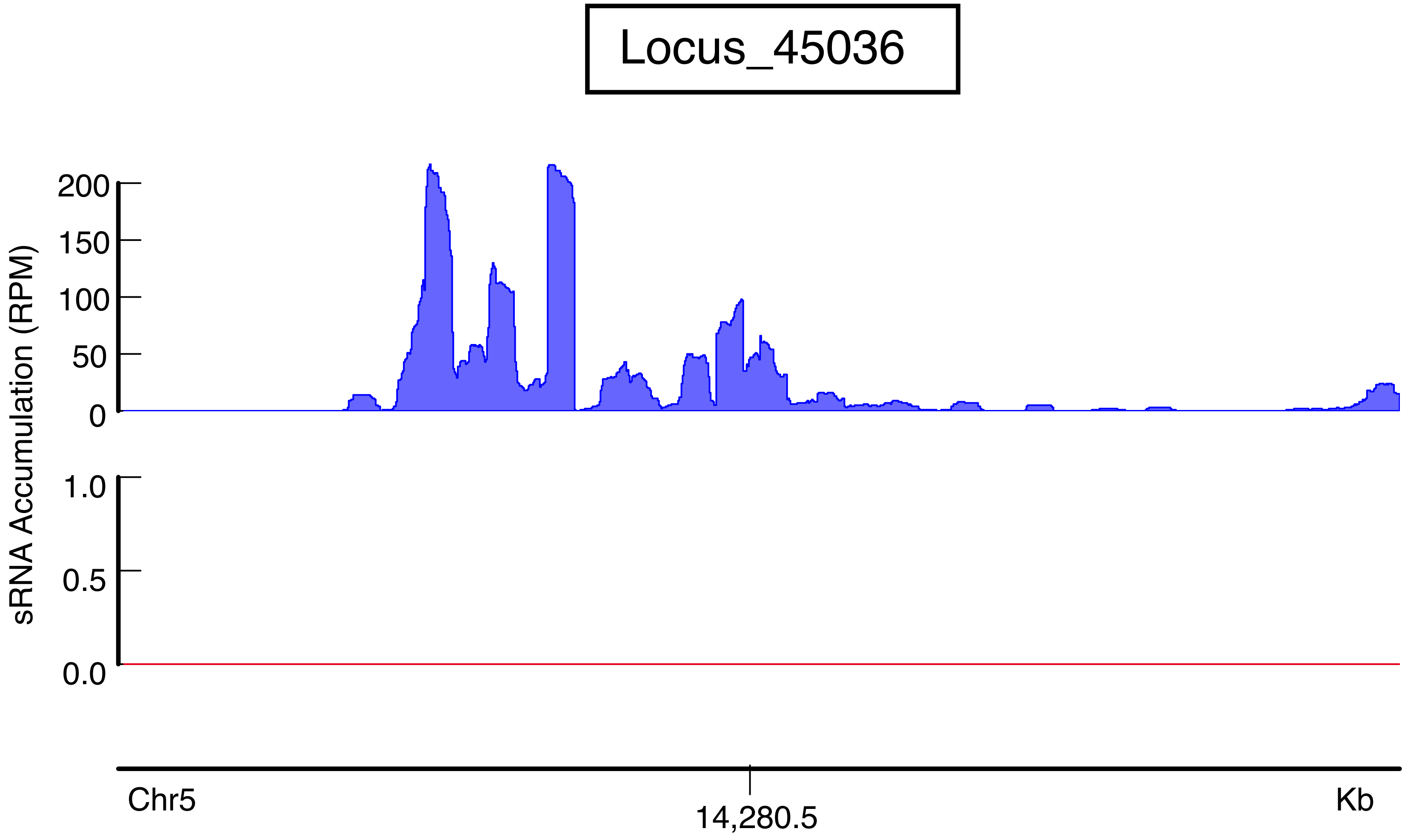

Figure S8 - Page 13

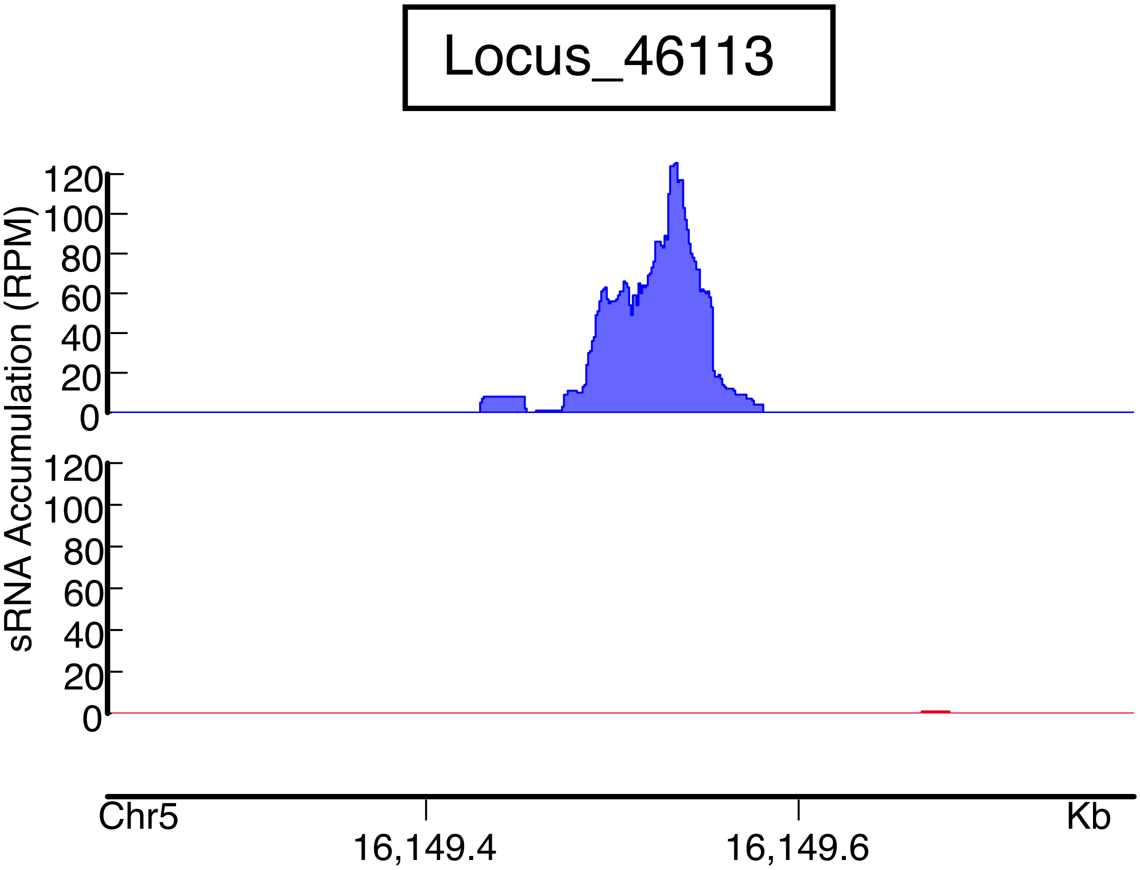

Figure S8 - Page 14

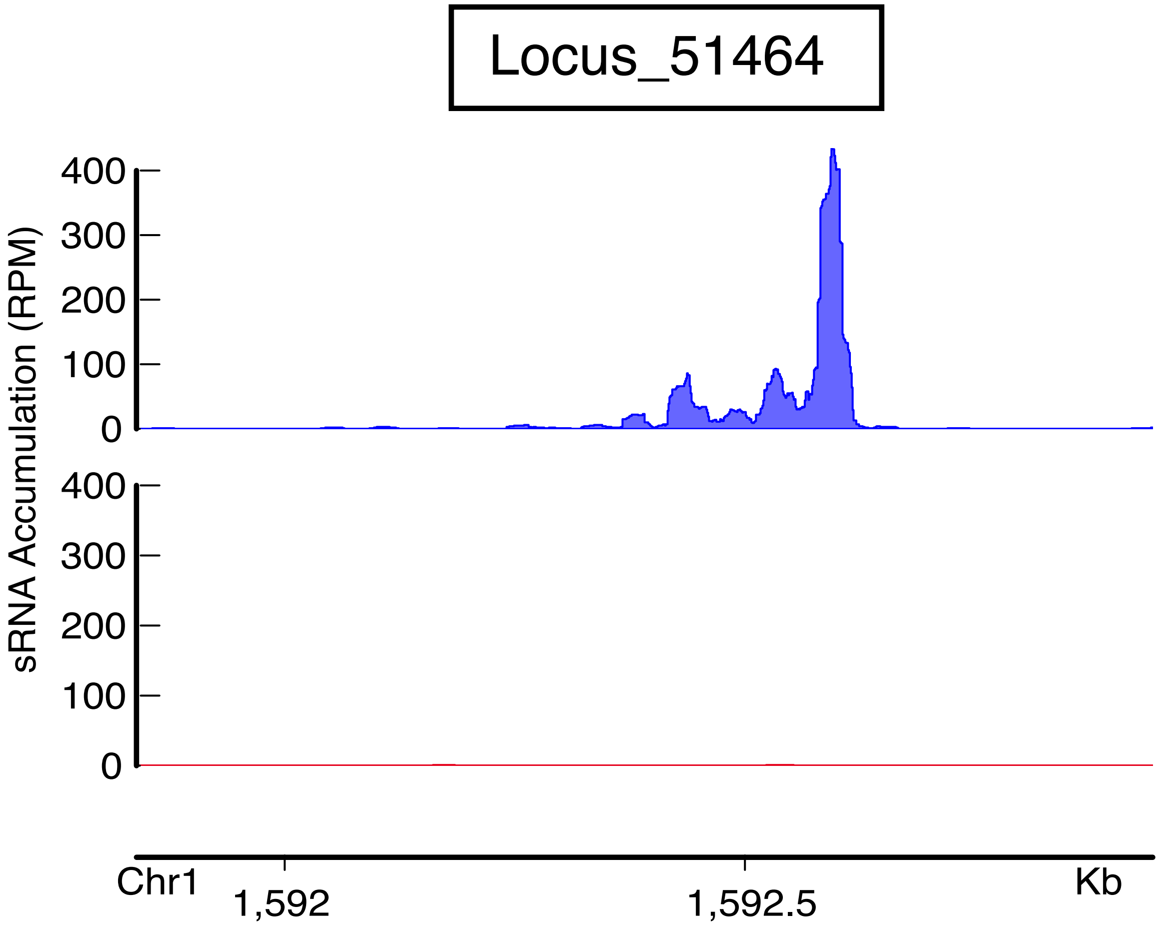

Figure S8 - Page 15

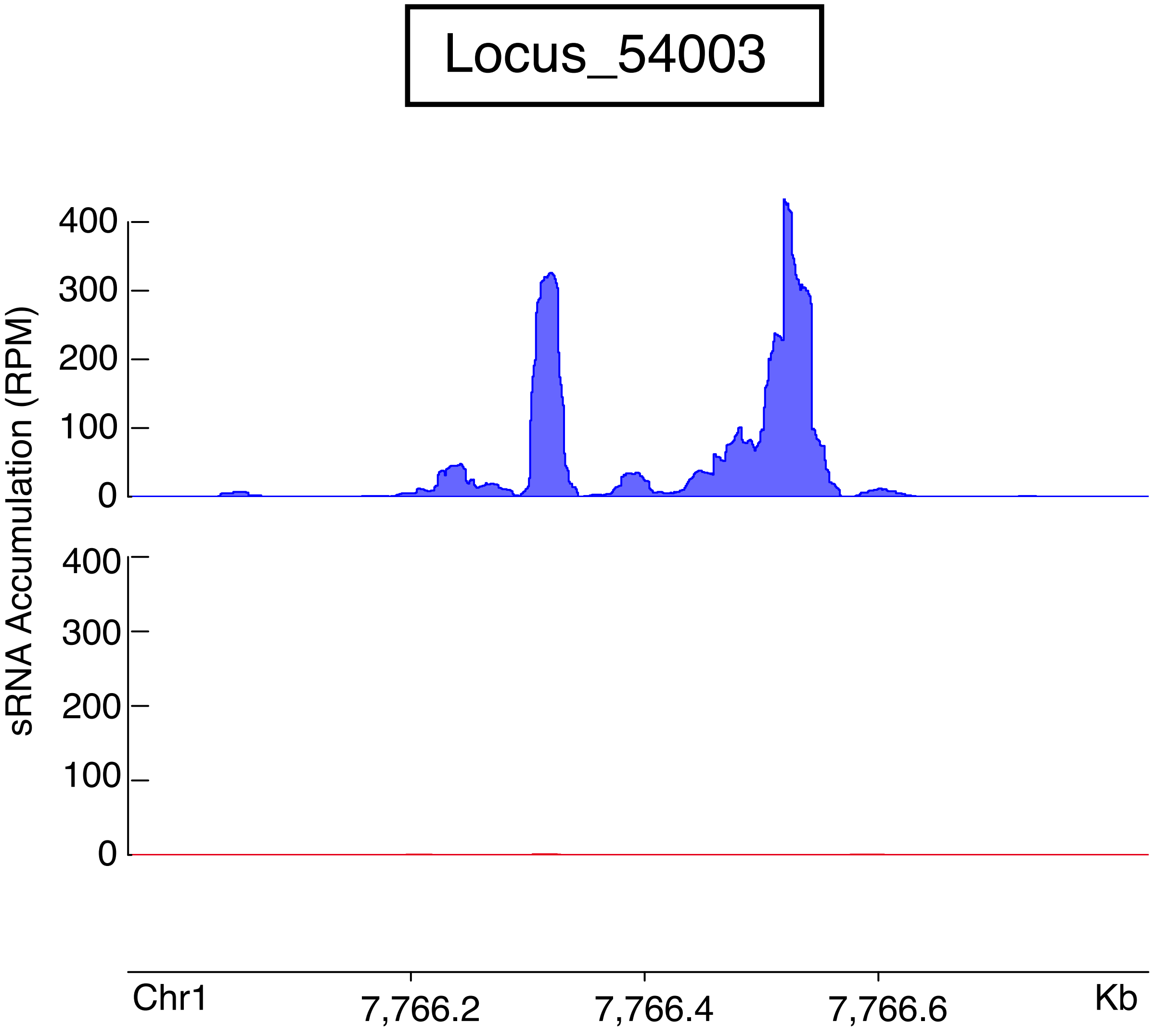

Figure S8 - Page 16

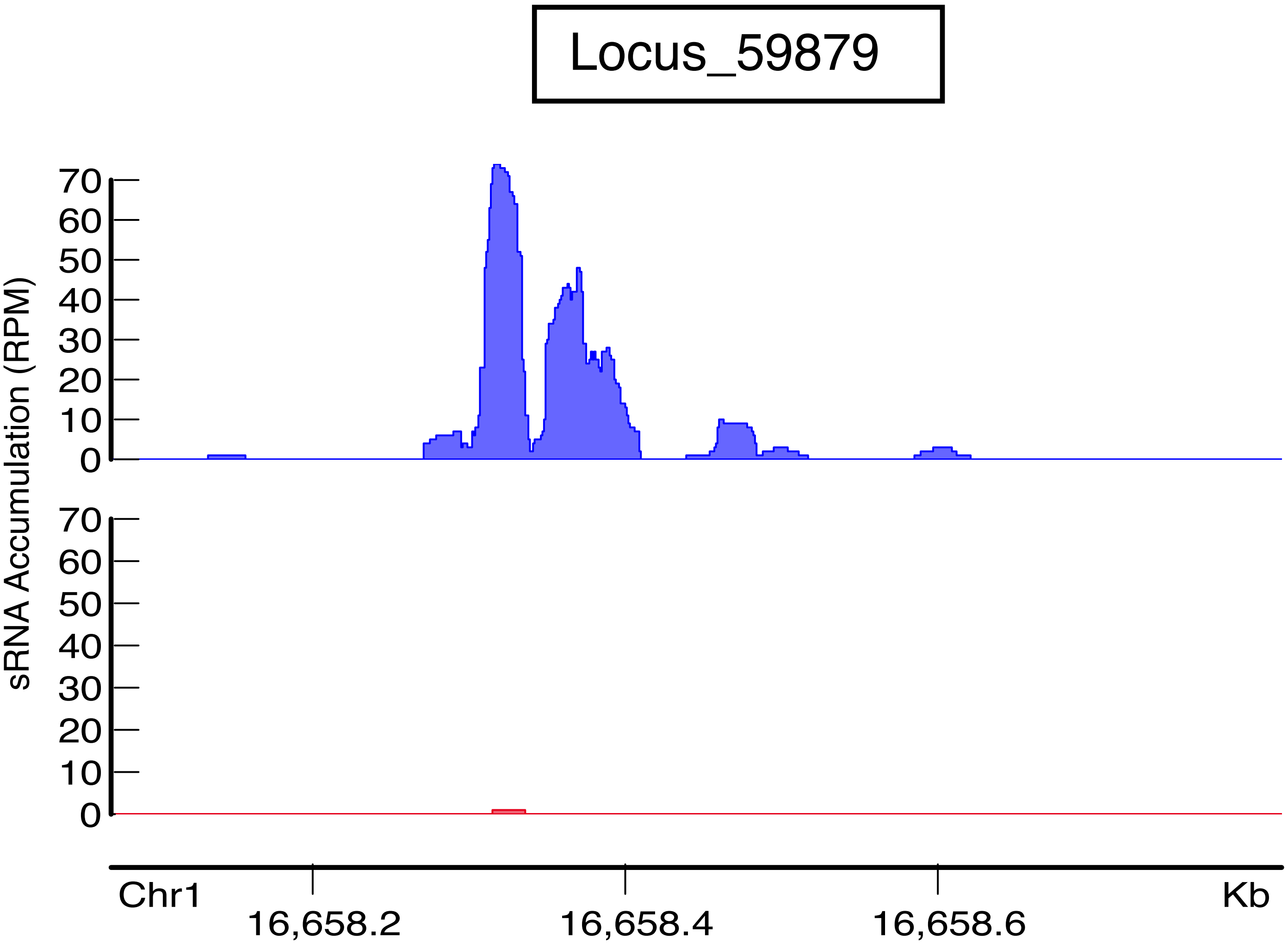

Figure S8 - Page 17

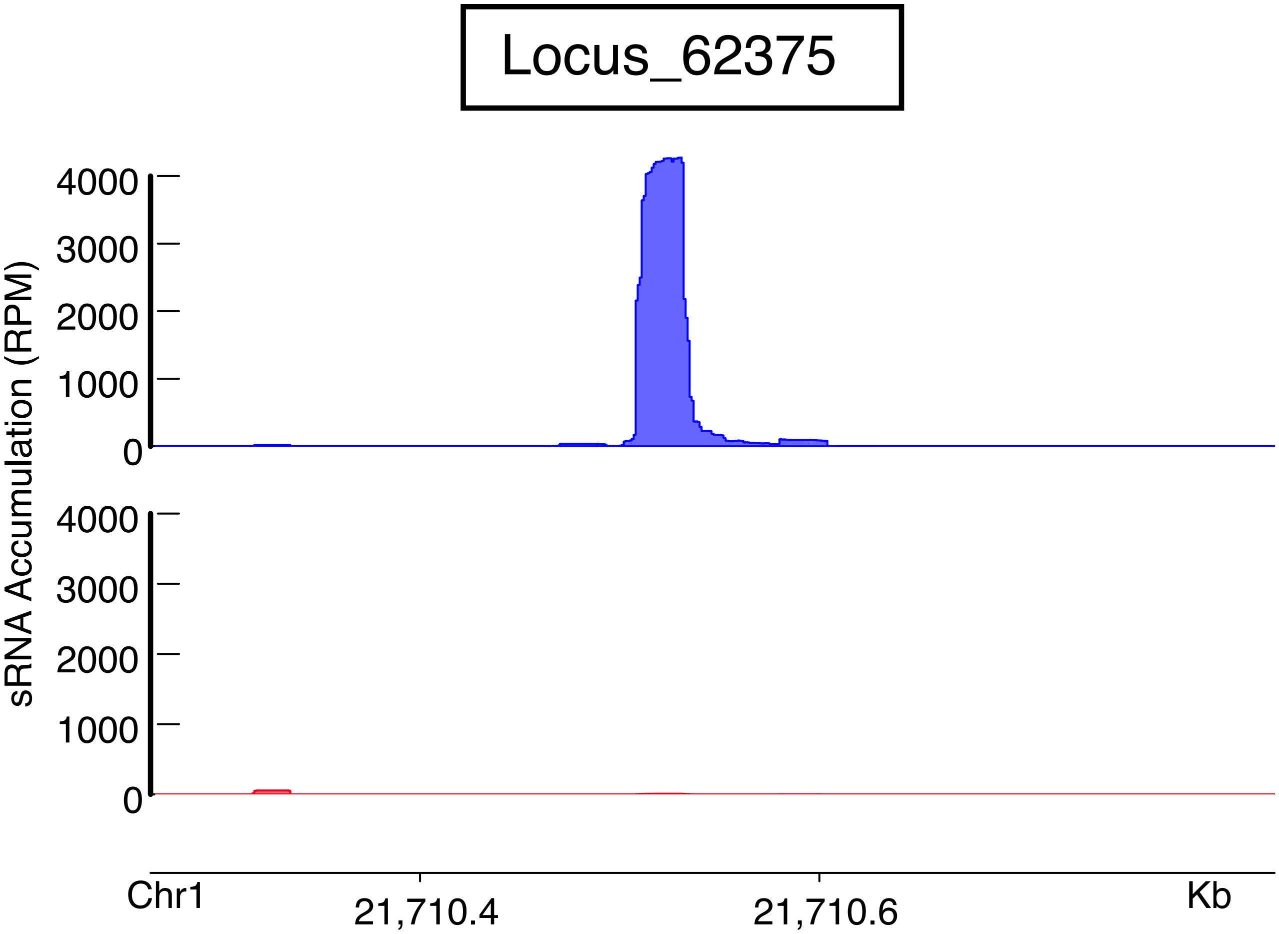

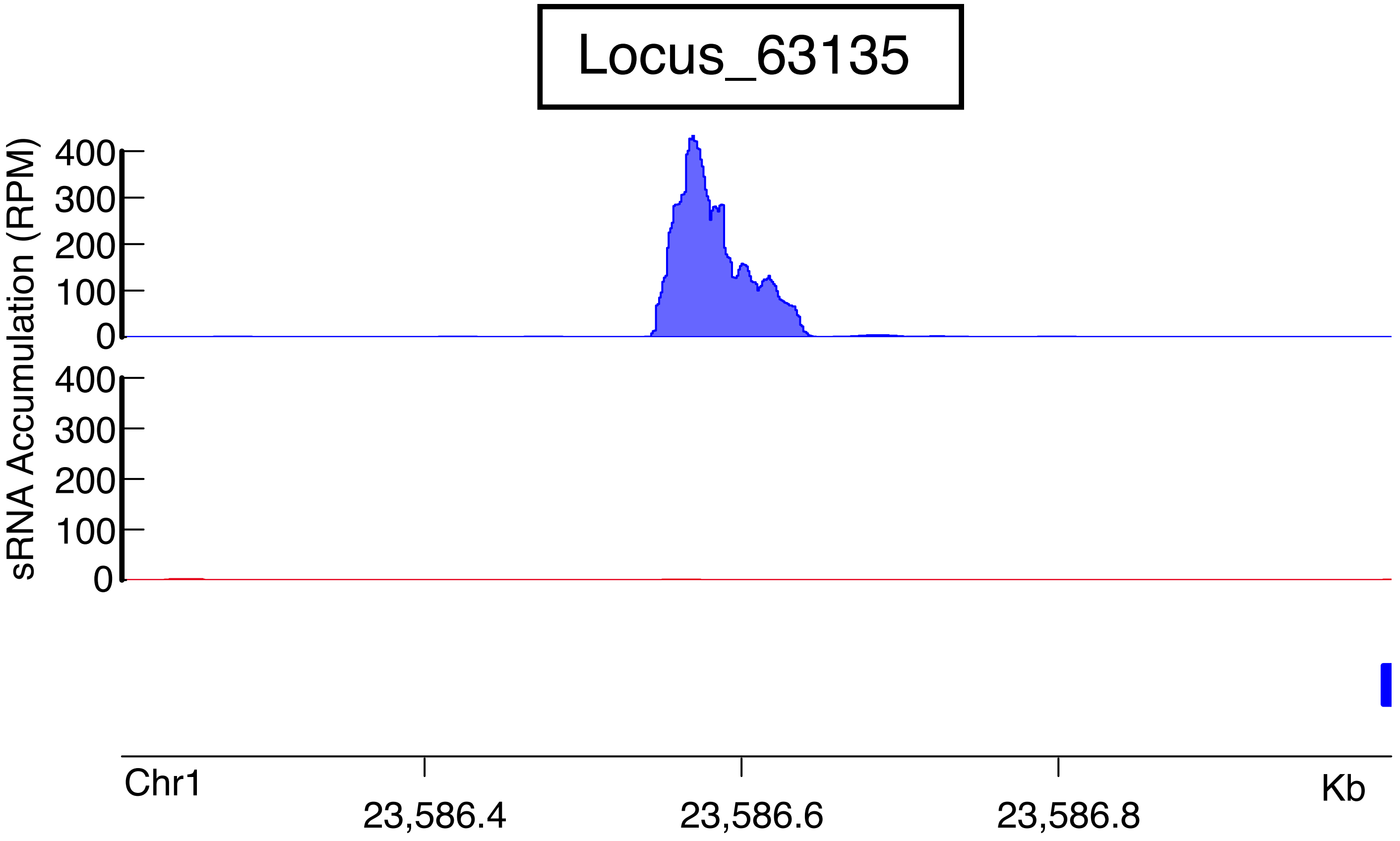
Locus_63135
sRNA Accumulation (RPM)
400
300
200
100
0
400
300
200
100
0
Chr1
23,586.4
23,586.6
23,586.8
Kb

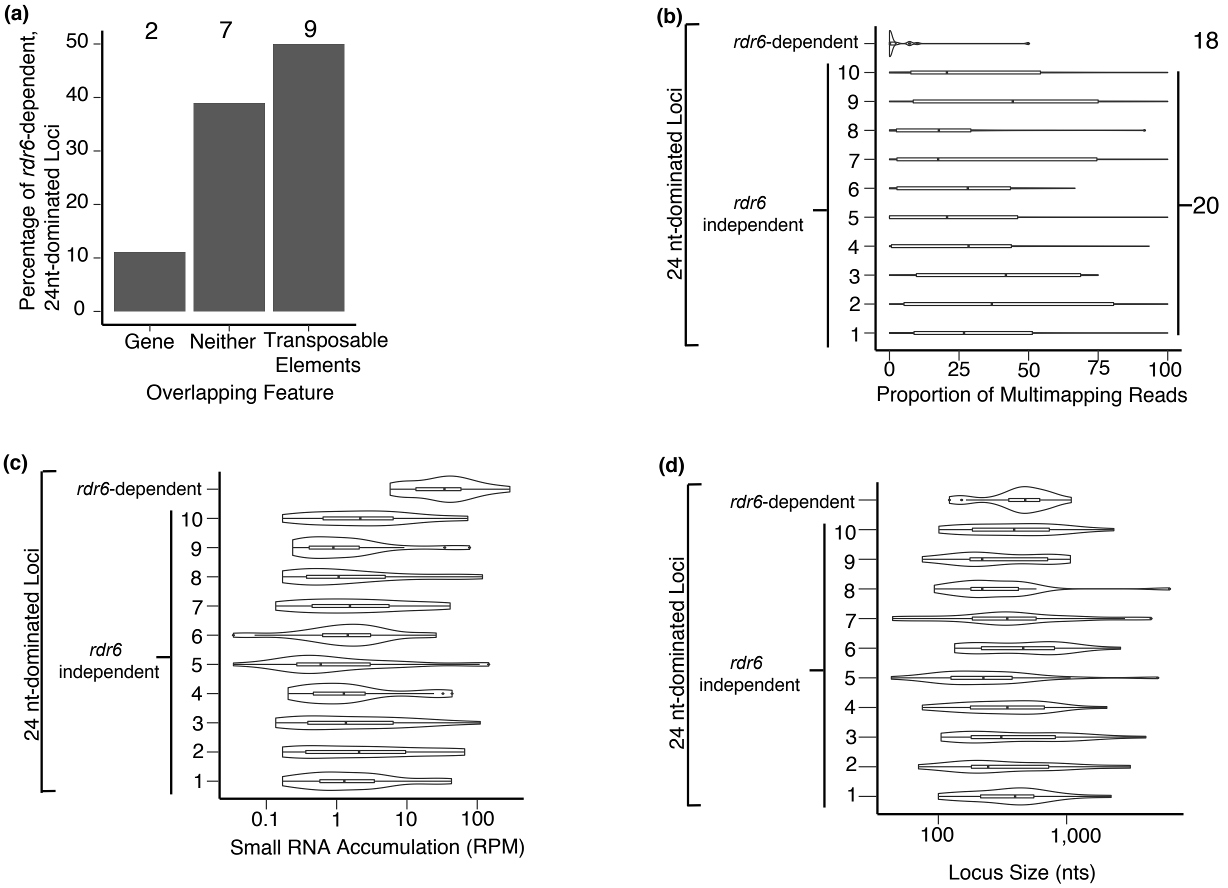

Figure S9. rdr6-dependent, 24 nt-dominated loci' characteristics compared to other 24nt-dominated loci.

(a) The overlap between rdr6-dependent, 24 nt-dominated loci with genes and transposons was calculated as in Figure 3a. Numbers at the top indicate the count in each category.

(b) The proportion of multi-mapping reads produced at rdr6-dependent, 24 nt-dominated loci compared to other 24 nt-dominated loci. Numbers at the top indicate the count in each category.

(c) Same as panel b except showing small RNA accumulation (in RPM). Amount in each category is the same as panel b.

(d) Same as panel b except showing length (in nts). Amount in each category is the same as panel b.

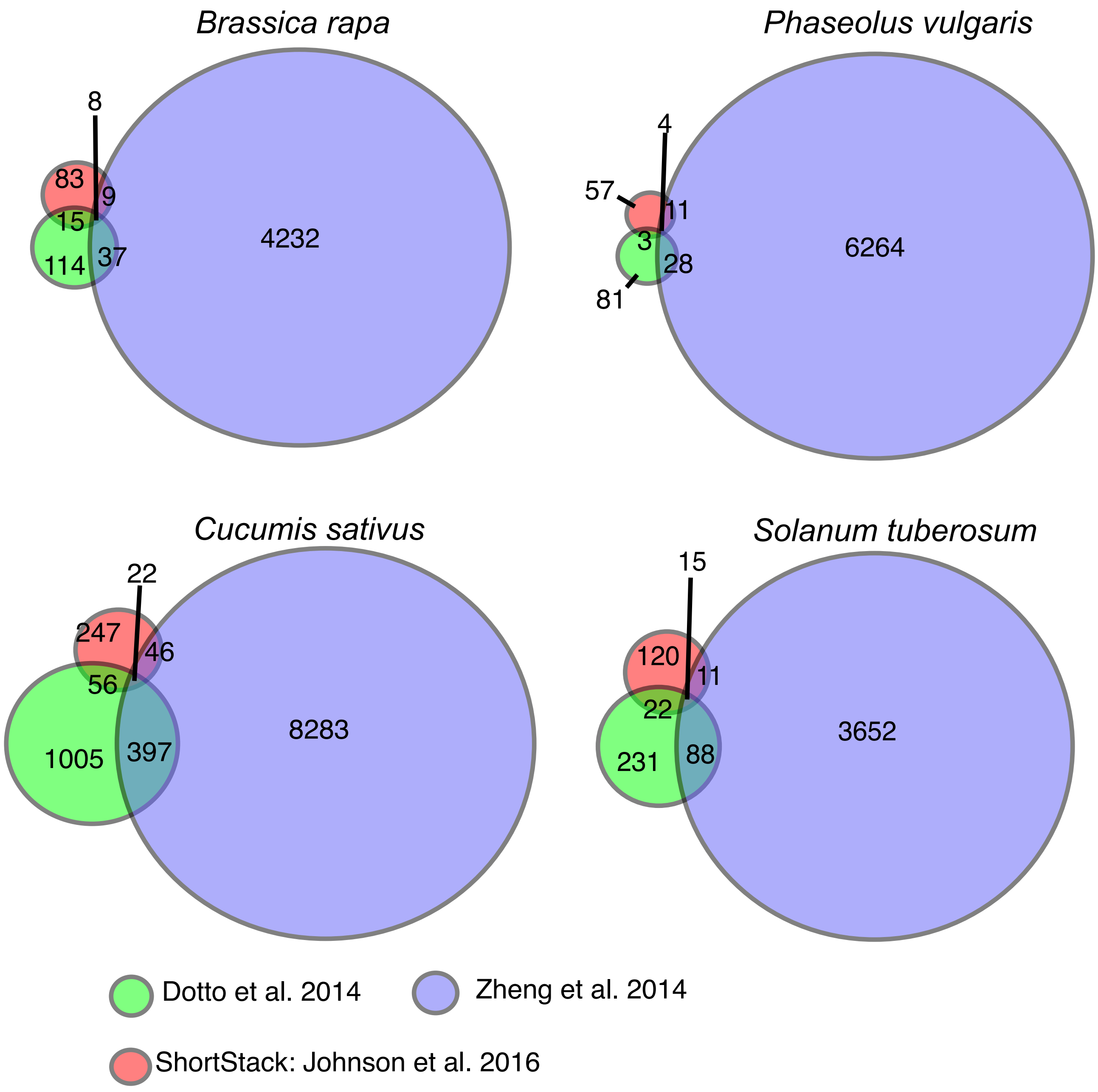

Figure S10. Several 24 nt-dominated small RNA loci pass PHAS-detection algorithms in four other eudicots.
Venn diagram shows numbers of 24 nt-dominated loci that were called 'phased' by the indicated algorithms. Species examined is shown above the graphs.

Table S1. List of 25 known 21 nt *PHAS* loci in *A. thaliana.*

Chr.	Start	Stop	Locus Name	Source
Chr2	11721539	11722468	*TAS1a*	(Vazquez et al., 2004)
Chr1	18549204	18550042	*TAS1b*	(Allen et al., 2005)
Chr2	16537288	16538277	*TAS1c*	(Allen et al., 2005)
Chr2	16539384	16540417	*TAS2*	(Allen et al., 2005)
Chr3	5861491	5862437	*TAS3a*	(Montgomery et al., 2008)
Chr5	20134200	20134786	*TAS3b*	(Howell et al., 2007)
Chr5	23394005	23394500	*TAS3c*	(Howell et al., 2007)
Chr3	9415004	9422587	*TAS4*	(Rajagopalan et al., 2006)
Chr1	23299057	23300958	*PPR-At1g62910*	(Ronemus et al., 2006)
Chr1	23412730	23415149	*PPR-At1g63130*	(Ronemus et al., 2006)
Chr1	23306534	23308683	*PPR-At1g62930*	(Ronemus et al., 2006)
Chr1	23387631	23390816	*PPR-At1g63080*	(Ronemus et al., 2006)
Chr1	23507320	23509053	*PPR-At1g63400*	(Ronemus et al., 2006)
Chr1	23419396	23421579	*PPR-At1g63150*	(Ronemus et al., 2006)
Chr1	23385324	23387167	*PPR-At1g63070*	(Ronemus et al., 2006)
Chr1	23489840	23491519	*PPR-At1g63330*	(Ronemus et al., 2006)
Chr1	23176930	23179248	*PPR-At1g62590*	(Ronemus et al., 2006)
Chr5	15555156	15558732	*TIR-NBS-LRR-At5g38850*	(Howell et al., 2007)
Chr1	4368760	4371293	*AFB3*	(Si-Ammour et al., 2011)
Chr5	16638370	16641728	*ATCHX18*	(Howell et al., 2007)
Chr1	17886098	17892586	*AGO1*	(Axtell et al., 2006)
Chr3	23273116	23276375	*TIR1*	(Si-Ammour et al., 2011)
Chr4	1404887	1407139	*AFB1*	(Si-Ammour et al., 2011)
Chr3	9867845	9870640	*AFB2*	(Si-Ammour et al., 2011)
Chr5	15757717	15758109	*SLG*	(Chen et al., 2007)
Chr4	8380848	8383496	*CC-NBS-LRR-At4g14610*	(Zhai et al., 2011)
Chr4	8146345	8152131	*MET2*	(Chen et al., 2010)

Reference Cited (Table S1)

Allen, E., Xie, Z., Gustafson, A. M., & Carrington, J. C. (2005). microRNA-directed phasing during trans-acting siRNA biogenesis in plants. *Cell*, *121*(2), 207–221. https://doi.org/10.1016/j.cell.2005.04.004

Axtell, M. J., Jan, C., Rajagopalan, R., & Bartel, D. P. (2006). A two-hit trigger for siRNA biogenesis in plants. *Cell*, *127*(3), 565–577. https://doi.org/10.1016/j.cell.2006.09.032

Chen, H.-M., Chen, L.-T., Patel, K., Li, Y.-H., Baulcombe, D. C., & Wu, S.-H. (2010). 22-nucleotide RNAs trigger secondary siRNA biogenesis in plants. *Proceedings of the National Academy of Sciences of the United States of America*, *107*(34), 15269–15274. https://doi.org/10.1073/pnas.1001738107

Chen, H.-M., Li, Y.-H., & Wu, S.-H. (2007). Bioinformatic prediction and experimental validation of a microRNA-directed tandem trans-acting siRNA cascade in Arabidopsis. *Proceedings of the National Academy of Sciences*, *104*(9), 3318–3323. https://doi.org/10.1073/pnas.0611119104

Howell, M. D., Fahlgren, N., Chapman, E. J., Cumbie, J. S., Sullivan, C. M., Givan, S. A., & Carrington, J. C. (2007). Genome-Wide Analysis of the RNA-DEPENDENT RNA POLYMERASE6/DICER-LIKE4 Pathway in Arabidopsis Reveals Dependency on miRNA- and tasiRNA-Directed Targeting. *The Plant Cell*, *19*(3), 926–942. https://doi.org/10.1105/tpc.107.050062

Montgomery, T. A., Howell, M. D., Cuperus, J. T., Li, D., Hansen, J. E., Alexander, A. L., & Carrington, J. C. (2008). Specificity of ARGONAUTE7-miR390 Interaction and Dual Functionality in TAS3 Trans-Acting siRNA Formation. *Cell*, *133*(1), 128–141. https://doi.org/10.1016/j.cell.2008.02.033

Rajagopalan, R., Vaucheret, H., Trejo, J., & Bartel, D. P. (2006). A diverse and evolutionarily fluid set of microRNAs in Arabidopsis thaliana. *Genes & Development*, *20*(24), 3407–3425. https://doi.org/10.1101/gad.1476406

Ronemus, M., Vaughn, M. W., & Martienssen, R. A. (2006). MicroRNA-Targeted and Small Interfering RNA–Mediated mRNA Degradation Is Regulated by Argonaute, Dicer, and

RNA-Dependent RNA Polymerase in Arabidopsis. *The Plant Cell*, *18*(7), 1559–1574. https://doi.org/10.1105/tpc.106.042127

Si-Ammour, A., Windels, D., Arn-Bouldoires, E., Kutter, C., Ailhas, J., Meins, F., & Vazquez, F. (2011). miR393 and Secondary siRNAs Regulate Expression of the TIR1/AFB2 Auxin Receptor Clade and Auxin-Related Development of Arabidopsis Leaves. *Plant Physiology*, *157*(2), 683–691. https://doi.org/10.1104/pp.111.180083

Vazquez, F., Vaucheret, H., Rajagopalan, R., Lepers, C., Gasciolli, V., Mallory, A. C., & Crété, P. (2004). Endogenous trans-Acting siRNAs Regulate the Accumulation of Arabidopsis mRNAs. *Molecular Cell*, *16*(1), 69–79. https://doi.org/10.1016/j.molcel.2004.09.028

Zhai, J., Jeong, D.-H., Paoli, E. D., Park, S., Rosen, B. D., Li, Y., & Meyers, B. C. (2011). MicroRNAs as master regulators of the plant NB-LRR defense gene family via the production of phased, trans-acting siRNAs. *Genes & Development*, *25*(23), 2540–2553. https://doi.org/10.1101/gad.177527.111

Table S2. *A. thaliana* small RNA libraries used in this study

Accession Number	Genotype	3' Adapter (First 8 nts)	Source
GSM2825283	Wild-type Replicate 1	TGGAATTC	(Polydore & Axtell, 2018)
GSM2825284	Wild-type Replicate 2	TGGAATTC	(Polydore & Axtell, 2018)
GSM2825285	Wild-type Replicate 3	TGGAATTC	(Polydore & Axtell, 2018)
GSM2825286	*rdr1-1/2-1/6-15* Replicate 1	TGGAATTC	(Polydore & Axtell, 2018)
GSM2825287	*rdr1-1/2-1/6-15* Replicate 2	TGGAATTC	(Polydore & Axtell, 2018)
GSM2825288	*rdr1-1/2-1/6-15* Replicate 3	TGGAATTC	(Polydore & Axtell, 2018)
GSM1533527	Wild-type Replicate 1	TGGAATTC	(Groth et al., 2014)
GSM1533528	Wild-type Replicate 2	TGGAATTC	(Groth et al., 2014)
GSM1533529	Wild-type Replicate 3	TGGAATTC	(Groth et al., 2014)
GSM1533542	*dcl3* Replicate 1	TGGAATTC	(Groth et al., 2014)
GSM1533543	*dcl3* Replicate 2	TGGAATTC	(Groth et al., 2014)
GSM1533544	*dcl3* Replicate 3	TGGAATTC	(Groth et al., 2014)
GSM1845210	Wild-type Replicate 1	AGATCGGA	(Elvira-Matelot et al., 2016)
GSM1845211	Wild-type Replicate 2	AGATCGGA	(Elvira-Matelot et al., 2016)
GSM1845212	Wild-type Replicate 3	AGATCGGA	(Elvira-Matelot et al., 2016)
GSM1845222	*dcl2-1/3-1/4-2t* Replicate 1	AGATCGGA	(Elvira-Matelot et al., 2016)
GSM1845223	*dcl2-1/3-1/4-2t* Replicate 2	AGATCGGA	(Elvira-Matelot et al., 2016)
GSM1845224	*dcl2-1/3-1/4-2t* Replicate 3	AGATCGGA	(Elvira-Matelot et al., 2016)
GSM1087973	Wild-type Replicate 1	TCGTATGC	(Jeong et al., 2013)
GSM1087974	Wild-type Replicate 2	TCGTATGC	(Jeong et al., 2013)
GSM1087975	*dcl1-7* Replicate 1	TCGTATGC	(Jeong et al., 2013)
GSM1087976	*dcl1-7* Replicate 2	TCGTATGC	(Jeong et al., 2013)
GSM1377370	Wild-type Replicate 1	TGGAATTC	(Li et al., 2014)
GSM1377371	Wild-type Replicate 2	TGGAATTC	(Li et al., 2014)
GSM1377372	*nrpd1-3* Replicate 1	TGGAATTC	(Li et al., 2014)
GSM1377373	*nrpd1-3* Replicate 2	TGGAATTC	(Li et al., 2014)
GSM1377376	*rdr2-1* Replicate 1	TGGAATTC	(Li et al., 2014)

GSM1377377	*rdr2-1* Replicate 2	TGGAATTC	(Li et al., 2014)
GSM2102962	Wild-type Replicate 1	TGGAATTC	(Panda et al., 2016)
GSM2102963	Wild-type Replicate 2	TGGAATTC	(Panda et al., 2016)
GSM2102965	*rdr6-15* Replicate 1	TGGAATTC	(Panda et al., 2016)
GSM2102462	*rdr6-15* Replicate 2	TGGAATTC	(Panda et al., 2016)
GSM893112	Wild-type Replicate 1	CACTCGGG	(Lee et al., 2012)
GSM893113	Wild-type Replicate 2	CACTCGGG	(Lee et al., 2012)
GSM893114	Wild-type Replicate 3	CACTCGGG	(Lee et al., 2012)
GSM893115	*nrpb1-1 (nrpe)* Replicate 1	CACTCGGG	(Lee et al., 2012)
GSM893116	*nrpb1-1 (nrpe)* Replicate 2	CACTCGGG	(Lee et al., 2012)
GSM893117	*nrpb1-1 (nrpe)* Replicate 3	CACTCGGG	(Lee et al., 2012)
GSM1668899	Wild-type Replicate 1	TGGAATTC	(Zhai et al., 2015)
GSM1668905	Wild-type Replicate 2	TGGAATTC	(Zhai et al., 2015)

Reference List (Table S2)

Elvira-Matelot, E., Hachet, M., Shamandi, N., Comella, P., Sáez-Vásquez, J., Zytnicki, M., & Vaucheret, H. (2016). Arabidopsis RNASE THREE LIKE2 Modulates the Expression of Protein-Coding Genes via 24-Nucleotide Small Interfering RNA-Directed DNA Methylation. *The Plant Cell Online*, *28*(2), 406–425. https://doi.org/10.1105/tpc.15.00540

Groth, M., Stroud, H., Feng, S., Greenberg, M. V. C., Vashisht, A. A., Wohlschlegel, J. A., & Ausin, I. (2014). SNF2 chromatin remodeler-family proteins FRG1 and -2 are required for RNA-directed DNA methylation. *Proceedings of the National Academy of Sciences of the United States of America*, *111*(49), 17666–17671. https://doi.org/10.1073/pnas.1420515111

Jeong, D.-H., Thatcher, S. R., Brown, R. S. H., Zhai, J., Park, S., Rymarquis, L. A., & Green, P. J. (2013). Comprehensive Investigation of MicroRNAs Enhanced by Analysis of Sequence Variants, Expression Patterns, ARGONAUTE Loading, and Target Cleavage1[W][OA]. *Plant Physiology*, *162*(3), 1225–1245. https://doi.org/10.1104/pp.113.219873

Lee, T., Gurazada, S. G. R., Zhai, J., Li, S., Simon, S. A., Matzke, M. A., & Meyers, B. C. (2012). RNA polymerase V-dependent small RNAs in Arabidopsis originate from small, intergenic loci including most SINE repeats. *Epigenetics*, *7*(7), 781–795. https://doi.org/10.4161/epi.20290

Li, S., Vandivier, L. E., Tu, B., Gao, L., Won, S. Y., Li, S., & Chen, X. (2014). Detection of Pol IV/RDR2-dependent transcripts at the genomic scale in Arabidopsis reveals features and regulation of siRNA biogenesis. *Genome Research*, gr.182238.114. https://doi.org/10.1101/gr.182238.114

Panda, K., Ji, L., Neumann, D. A., Daron, J., Schmitz, R. J., & Slotkin, R. K. (2016). Full-length autonomous transposable elements are preferentially targeted by expression-dependent forms of RNA-directed DNA methylation. *Genome Biology*, *17*, 170. https://doi.org/10.1186/s13059-016-1032-y

Polydore, S., & Axtell, M. J. (2018). Analysis of RDR1/RDR2/RDR6-independent small RNAs in Arabidopsis thaliana improves MIRNA annotations and reveals unexplained types of short interfering RNA loci. *The Plant Journal: For Cell and Molecular Biology*, *94*(6), 1051–1063. https://doi.org/10.1111/tpj.13919

Zhai, J., Bischof, S., Wang, H., Feng, S., Lee, T., Teng, C., & Jacobsen, S. E. (2015). One precursor One siRNA model for Pol IV- dependent siRNAs Biogenesis. *Cell*, *163*(2), 445–455. https://doi.org/10.1016/j.cell.2015.09.032
