# Supplementary material for: Several phased siRNA annotation methods can frequently misidentify 24 nucleotide siRNA‐dominated PHAS loci
Source: Plant Direct. 2018 Dec 17;2(12):e00101. doi: 10.1002/pld3.101 (PMC6508839; doi:10.1002/pld3.101)
Supplement: Supplementary file 6 [file PLD3-2-e00101-s006.pdf]

> Dear Colleagues: Thank you for your time and constructive comments on our manuscript. We have read and responded to each of your comments and made many revisions to the manuscript. These revisions have undoubtedly improved our manuscript. Detailed responses and specific descriptions of the revisions are listed below, interspersed with your original comments. Our responses are in bold, blue-colored text, prepended by the > symbol.

The paper "Several phased siRNA annotation methods can produce frequent false positives for 24 nt RNA-dominated loci in plants" is a very interesting addition to the field especially because it highlights a recurring problem with prediction algorithms.
The manuscript is well structured, scientifically sound and the arguments are solid;

> Thank you for the positive comments!

I would like the following aspects to be clarified.

Major comments:
(1) I would like to see the title rephrased - as it stands it is not entirely clear what is the subject for "false positives"; the authors should clarify that the phasing is on a 24nt register and the phased loci are likely to be FPs.

> The title has been changed to " Several phased siRNA annotation methods can frequently misidentify 24 nucleotide siRNA-dominated *PHAS* loci ". (lines 1-2).

(2) The main criticism for the lack of clarity of the abstract (for a general audience) can be addressed by introducing the "phasing problem" gradually; the start of the abstract is perfect, after introducing the miRNAs and siRNAs continue along the same lines. E.g. "imprecisely processed" is too vague for readers not familiar with the field; next the role of miR 2275 is not clear - first I would like to see few words on the role of miRNAs in the production of tas loci, and then it would become clearer why the focus is on miR 2275

> A sentence that explains the role of miRNAs in phasiRNA biogenesis has been added to the abstract. (lines 14-16).

(3) I like the conservation analysis of miRNA 2275, however some elements of the analysis should be addressed
a. The phylo tree should be to scale and the branches should be used as proxy for speciation distances.

> This has been done, using timetree of life estimates for divergence times. See Figure 1. (~line 125).

Also the multiple sequence alignment would be very informative for the readers and would also reveal the location of mutations (along the mature miR/miR* or the stem and provide a discussion point on the weighted approach that emphasises more the high selection pressure on the mature fragments in contrast to the rest of the stem)

> We have done this; it is now included as Figure S2. (~ line 726).

b. Rows 136-140: it is not entirely clear whether you were looking for known miRNAs to trigger the 24-nt dominated locus.

> The sentence states "...we predicted whether or not any known A. thaliana miRNAs could target these three loci", so as we stated it is using known miRNAs. (now at lines 145-146).

To make this search exhaustive I suggest to look for putative new miRNAs using a function first approach i.e. identify the putative sequence of a targeting miRNA, then match the read on the genome looking for suitable loci with a hairpin-like secondary structure. The fragment may or may not be present in sequencing libraries - depending on the sequencing bias- but its presence can always be tested using a wet-lab validation approach e.g. northern blot

> We thank the reviewer for the suggestion but we have elected to not pursue this analysis for two reasons: 1) In the end, we conclude that these loci are not truly phased anyway; that's the major conclusion of the whole study. 2) While we acknowledge there could be biases in sRNA-seq libraries that would obfuscate discovery of certain small RNAs, the approach of reverse-searching for siRNAs or miRNAs in absence of any sRNA-seq data has a dismal history of false positives in the previous literature.

c. Figure 2 (page 9). No info was presented on whether the loci were distinct/unique (i.e. if some loci are substrings of other, then these should not be double-counted);

> All small RNA loci have distinct, non-overlapping genomic coordinates so there was no double-counting. We have clarified this point in the Methods section. (line 447).

subplot (b) is misleading - I assume the numbers at the top of the histogram are the number of loci. It is incorrect to compare 3 loci with 31k.

> We've replaced the analysis in Figure 2b with one that samples multiple, small-n (20 loci each) cohorts from the 'Not *PHAS*' set. (~ line 156). Qualitatively, the conclusion is that same: The 3 'passing' loci have a similar percentage of TE overlaps to the non-passing set. We've modified the methodology in Figure 3 similarly. (~ line 219).

For (c) what was the noise level for these loci i.e. do the fold changes make sense or are they solely derived from low-level variation

> These are the DESeq2 estimated mean log2-fold changes. As indicated on the figure, cells highlighted in red are significant changes (FDR 0.1).(~ line 157).

(4) General comment: you are using the terms locus and cluster interchangeably - please choose one term and be consistent throughout. I prefer the term locus since this reflects the biological function of a location in the genome. I know that the term cluster has been used for some time, however it is misleading in the current machine-learning environment and be avoided, unless it refers to the identification of patterns in an unsupervised manner.

> Thank you, we agree and have changed to the term locus / loci throughout the revised manuscript.

(5) Row 376 avoid using blast for drawing conclusions on sRNAs - the algorithm was designed to work on longer sequences. Other tools such as bowtie or patman are more appropriate for this task.

> We have re-done the search using bowtie (allowing up to two mismatches) and have found largely the same results: We lost two hits (*Musa accuminata* (Banana) and *Brachypodium stacei*) but found two others (*Brachypodium distachyon* and *Malus domestica*). The revision uses the bowtie method.

(6) Row 402: "200 nts" the secondary structures depend on the input (the quality and length), a search of the best possible secondary structures using incremental windows is more suitable (and will provide a clearer answer). In addition, I recommend testing some locus identification algorithms like segmentseq (Hardcastle et al 2010) or colide (Mohorianu et al 2013) and use the pattern characterisation from these tools to refine your results.

> Thank you for the suggestion. We did not pursue segmentseq or colide because this particular analysis did not involve analysis of genome-aligned sRNA-seq data. Instead, this analysis was merely predicting RNA secondary structures from genomic regions around bowtie hits to miR2275. Using mFold, we have manually investigated subsections of the arbitrary +/-200 nt genomic windows and confirmed that the predicted hairpins we report are those with the minimum deltaG. We find that the mFold-predicted secondary structures are quite robust regardless of how much or how little flanking sequence is included in the secondary structure predictions.

(7) For the plots of secondary structures ... first include the name of the miRNA being plotted, second try to either provide a justification for weird structures (e.g. C. sinensis) or exclude them.

> Fig S1 has been modified to include microRNA names. We don't believe there are any predicted secondary structures that are too "weird" although of course this is a judgment call. (line 712-717).

(8) Row 732 - for the radial plots, what do the numbers on the y-axis indicate? Are these abundances (linear or logarithmic scale)? If the abundance scale is linear, provide some justification that these are not in the noise range.

> The numbers on the radial plots of Fig S6 are percentages, plotted on a linear scale. This is noted in the figure legend for Fig S6 (lines 759-763). As we describe in the main text (lines 150-155), the first three plots (our 3 'false positive' loci) indeed do seem to be just noise .. no strongly predominating phase register and little reproducibility between different libraries. *TAS2* is included as a positive control.

(9) Row 751 : the presence plots seem to be artificially elongated; I would like to see a comparison with other methods or a justification for the length of these loci.

> We agree that the boundaries of our siRNA loci are set by automated detection and that alternative methods of locus discovery might set the locus boundaries differently. The exact method of locus-finding used by our ShortStack script are now described explicitly in the main text: "*All distinct genomic intervals containing one or more primary sRNA-seq alignments within 75 nts of each other were obtained, and then filtered to remove loci where the total sRNA-seq abundance with a locus was less than 0.5 reads per million.*" (lines 444-447). We agree that an in-depth comparison with other small RNA locus-finding methods would be interesting, but we contend that it is quite a bit beyond the scope of this study: Implementing multiple locus-finding techniques amounts to re-doing every other downstream analysis for our entire study. We have added text in several places of the revision discussing the fact that locus-boundary settings may also influence false-discoveries of phased siRNA loci, especially because our 'false' ones were very long (Fig 3C).

Minor comments:
(1) row 45: the phrase "single-stranded, stem and loop" should be replaced wit "single stranded RNA with a hairpin like secondary structure"

> Done. (line 47).

(2) row 51: some words are missing "biogenesis of hc-siRNAs begins with the transcription of TEs"; also hc-siRNAs are not restricted to TEs, they can also derive/target from promoters and other siRNA loci along the genome

> Agreed, rephrased. (lines 51-54)

(3) row 61: a citation is necessary at the end of the sentence.

> Done. (lines 68-70).

(4) Row 201: the lengths of these loci are worrying and I would like to see a full

description of reads properties and a justification that these loci are above the noise level.

> Please see response to your Major Comment #9 above. In regard to this figure (Figure 3c), whatever one thinks about the method by which the boundaries of the loci were determined, it is the same method applied consistently, so the comparison across the three classes of loci seems valid. Our 'false positive' loci are clearly quite long relative to the bulk of 24 nt siRNA loci and we've added that to the discussion. (lines 350-353).

(5) Rows 215-216: please refine the captions.

> Done (refers to Figure 3; lines 221-231).

(6) Row 256: the rationale is not entirely clear - do clarify it.

> Done (lines 270-272).

(7) Row 276: as remarked earlier the comparisons presented in subplot (a) are not meaningful. You could try a subsampling approach to answer the question: "how often would we get a similar approach when 8/4 loci are selected at random"; however, given the low number of loci, I doubt that the subsampling would be stable in itself.

> Refers to Figure 5. We've re-done the analysis with the suggested approach, reflected in the new Figure 5 (~ line 288). Qualitatively, the results are much the same: No obvious biases for gene/TE overlaps or for multi-mapping reads, but clear bias toward long clusters with abundant siRNAs.

(8) Row 846: this is only a remark - it is very useful to see the information on the sequencing adapter. Due to the strong effect of sequencing bias, I would not combine libraries built with different technologies - each sequencing adapter reveals part of the sRNA population; the sequencing libraries are not wrong, just incomplete.

> 3' adapter sequences are now listed in Table S2. We agree that adapter-generated sequence bias is real. We have not combined libraries made by different methods for differential expression analyses. All of the differential expression analysis was between wild-type and mutant libraries made by the same lab with the same methods; there weren't cross-adapter comparisons made with respect to differential expression.

Reviewer #2:

The authors addressed a very important yet often overlooked aspect in exploring small RNA sequencing data. The analyses are well designed and solidly performed. The manuscript is already in good shape so I just have mostly minor comments.

> Thank you for the positive comments!

Major comment:
1. Since the algorithm from Dotto et al. (2014) paper is studied extensively in this manuscript, and the authors reported frequent false-positives for 24 nucleotide loci, I wonder if the author could also evaluate and comment on the 22-nt phased siRNA loci described in the Dotto et al. (2014) paper - "With a P-score threshold (P{greater than or equal to}25) that has been shown to identify 7 of the 8 TAS loci in Arabidopsis [21], [31], we identified 16 phased 21-nt siRNA clusters, 102 phased 22-nt siRNA clusters, and 8 phased 24-nt siRNA clusters". This may suggest that, in addition to 24-nt, highly expressed 22-nt sRNA clusters are also subject to frequent false-positives.

> Thank you. We added a generic comment into the discussion that even for 21- or 22-nt dominated *PHAS* loci, caution should be taken (lines 403-405). However, we didn't want to specifically re-examine the 22nt annotations made by Dotto et al. both because our focus here is on 24 nt loci and because we do not wish to directly criticize any specific prior work.

Minor Comments:

1. line 108, Ten eudicots had potential miR2275 homologs
According to Figure 1, it should be eleven eudicots, not ten eudicots, that had potential miR2275 homologs based on sequence similarity.

> Thank you for catching this. We've revised the methods on this part according to a comment from the other reviewer, so the correct number is now actually 12. It's correctly stated now. (line 112).

2. line 131, Reasonable cut-offs for PHAS loci detection
1) The pipeline of each algorithm used here were not clarified. Considering that each algorithm relies steps of preprocessing, simply employing formula might make mistakes.

> We applied each algorithm to exactly the same data (same alignments from the same loci), so all pre-processing was the same. We've clarified that in the methods section (lines 452-457).

2) Can three libraries determine the right cut-offs? how about the changes of phase score distributions and corresponding cut-offs with other three libraries or with increasing numbers of libraries.

> Yes, the determination of cutoffs is ultimately a judgement call, no matter how many different libraries one looks at. However, the phase score distributions at known loci are rather consistent with a greater number of libraries. Figure S4 (~

line 741) has been added to demonstrate this.

3. line 716, Fig S2, Determination of phase score cutoffs
1) (b) formula comes from Dotto et al. (2014) not Zheng et al. (2014)
2) (c) formula comes from Zheng et al. (2014) not Dotto et al. (2014)

> Thank you for catching that. Corrected. (Now Figure S3; lines 733-740).

3) Zheng's paper reports two formulas to calculate P-value and Phase Score, and further calculate the multiple test corrected P-values, while this paper only used the uncorrected P-value and abandoned the Phase Score, why?

> We wanted to use only one metric for each of the different algorithms being compared. To keep it simple, we used the P-value from Zheng's method instead of the phase score. We did not correct for multiple testing because we wished to directly compare to the other two methods (which do not produce p-values and thus can't easily be corrected for multiple testing). We added text to the methods section of the revised manuscript to explain this (lines 438-440).

4. line 133, well-known 21 nt PHAS loci were analyzed (Figure S2)
The known 21nt PHAS loci in Table S1 should include TAS3b and TAS3c, in addition, the corresponding phase scores or p-values of known 21nt PHAS loci data cannot be seen

> *TAS3b* and *TAS3c* have been added to Table S1 (~ line 818). Phase scores and p-values have been included in Dataset S4.

5. line 139, it's possible that other siRNAs might target them and initiate siRNA phasing.
Is there any siRNA known to target these clusters?

> In the end we concluded that these are not truly phased anyway based on several other lines of evidence, so we did not perform this analysis which would be complicated in practice, and not supported by precedent (all known 24 nt phasiRNAs are triggered by a microRNA, not siRNAs).

6. line 169, these three loci is down-regulated in nrpd1-3 nrpd, and rdr2
1) Why the accumulation of the three clusters are not down regulated in dcl3 mutant in Figure 2c?

> Based on previous work, we suspect that other *DCL2* and *DCL4* partially compensate for siRNA accumulation in *dcl3* single mutants. We've added this point in the revised text. (lines 184-186).

2) Is there any cluster identified by either PHAS test algorithms have different pattern of hc-siRNAs?

> Not that we observed, although we are focused only on the loci that 'passed' all three methods and did not interrogate loci that failed to pass all three methods.

7. line 147, Figure 2e
How about the size distribution of other three TAS loci in Figure2e, such as TAS1C, TAS3C and TAS4?

> *TAS1C* has been added to the revised figure 2e. *TAS3C* and *TAS4* were had little to no sRNA accumulation in our datasets, so they remain omitted.

8. line 361, "phasiRNAS" should be changed to "phasiRNAs".

> Done. (now line 377).
